# Macromolecular Design for Oxygen/Nitrogen Permselective Membranes—Top-Performing Polymers in 2020—

**DOI:** 10.3390/polym13173012

**Published:** 2021-09-06

**Authors:** Jianjun Wang, Zhichun Shi, Yu Zang, Hongge Jia, Masahiro Teraguchi, Takashi Kaneko, Toshiki Aoki

**Affiliations:** 1 Key Laboratory of Polymeric Composition Material of Heilongjiang Province, College of Materials Science and Engineering, Qiqihar University, Wenhua Street 42, Qiqihar 161006, China; 02900@qqhru.edu.cn (J.W.); zangyu@qqhru.edu.cn (Y.Z.); 01723@qqhru.edu.cn (H.J.); 2 Technology Innovation Center of Industrial Cannabis Processing of Heilongjiang Province, College of Chemistry and Chemical Engineering, Qiqihar University, Wenhua Street 42, Qiqihar 161006, China; 02897@qqhru.edu.cn; 3 Department of Chemistry and Chemical Engineering, Graduate School of Science and Technology, Niigata University, Ikarashi 2-8050, Nishi-Ku, Niigata 950-2181, Japan; teraguti@eng.niigata-u.ac.jp (M.T.); kanetaka@gs.niigata-u.ac.jp (T.K.)

**Keywords:** macromolecular design, oxygen/nitrogen separation, polymer membranes, top-performing polymers, upper bounds, oxygen permeability, oxygen permselectivity

## Abstract

Oxygen/nitrogen permselective membranes play particularly important roles in fundamental scientific studies and in a number of applications in industrial chemistry, but have not yet fulfilled their full potential. Organic polymers are the main materials used for such membranes because of the possibility of using sophisticated techniques of precise molecular design and their ready processability for making thin and large self-supporting membranes. However, since the difference in the properties of oxygen and nitrogen gas molecules is quite small, for example, their kinetic diameters are 3.46 Å and 3.64 Å, respectively, the architectures of the membrane macromolecules should be designed precisely. It has been reported often that oxygen permeability (*P*_O_2__) and oxygen permselectivity (α = *P*_O_2__/*P*_N_2__) have trade-off relationships for symmetric membranes made from pure polymers. Some empirical upper bound lines have been reported in (ln α − ln *P*_O_2__) plots since Robeson reported an upper bound line in 1991 for the first time. The main purpose of this review is to discuss suitable macromolecular structures that produce excellent oxygen/nitrogen permselective membranes. For this purpose, we first searched extensively and intensively for papers which had reported α and *P*_O_2__ values through symmetric dense membranes from pure polymers. Then, we examined the chemical structures of the polymers showing the top performances in (ln α − ln *P*_O_2__) plots, using their aged performances. Furthermore, we also explored progress in the molecular design in this field by comparing the best polymers reported by 2013 and those subsequently found up to now (2020) because of the rapid outstanding growth in this period. Finally, we discussed how to improve α and *P*_O_2__ simultaneously on the basis of reported results using not only symmetric membranes of pure organic polymers but also composite asymmetric membranes containing various additives.

## 1. Introduction

### 1.1. Significance of Oxygen/Nitrogen Permselective Membranes

Gas separation membranes are very important and have a promising future not only because of their scientific interest to chemists and scientists but also for their practical applications such as removal of contaminants and purification of resources important to chemical and materials engineers [1,2,3,4,5,6,7,8]. Therefore, intensive and continuous studies on gas separation membranes have been carried out for many years that have resulted in some practical uses. In particular, oxygen/nitrogen permselective membranes are very important in both the industrial and medical fields. For example, they can be used to treat seriously ill patients with a pulmonary disease like COVID-19. However, the performances of gas separation membranes, especially for separating oxygen and nitrogen, are not as good as desired because their sizes and properties are very similar. For example, the kinetic diameters of an oxygen molecule and a nitrogen molecule are 3.46 Å and 3.64 Å, respectively, and this size difference is extremely small (0.18 Å). As materials for such membranes, soluble organic polymers are popular and useful because they can be fabricated very easily and economically as dense thin membranes without any void defects through which gas molecules can pass freely without any selectivity.

### 1.2. Requirements for Polymers as Oxygen/Nitrogen Permselective Membranes

There are mainly two requirements for polymers as permselective membranes for a gas mixture of oxygen/nitrogen as follows [1].

(1) high processability, mechanical strength, and stability
(2) high oxygen permselectivity, that is, a high oxygen permeability coefficient (*P*_O_2__ (barrer)) and high oxygen separation factor (α = *P*_O_2__/*P*_N_2__)

The oxygen permeability coefficient (*P*_O_2__ (barrer)) is the quantity (volume: cc(STP)) of oxygen gas molecules permeated through a polymer membrane normalized by the membrane area (cm^2^), membrane thickness (cm), permeation time (s), and pressure difference applied (driving force) (cmHg). The unit, barrer, is defined by 1 barrer = 1 × 10^−10^ cc (STP) cm cm^−2^ s^−1^ cmHg^−1^, where STP means a standard temperature (25 °C) and pressure (1 atm). Since this is a characteristic value for each polymer architecture, this value basically depends on the macromolecular structure. However, different *P*_O_2__ values for polymers with the same first-order structures (chemical structures) were often reported because they could have different high-ordered structures (solid-state structures or membranes structures) even if the polymers had the same macromolecular structures.

There are two main trade-off problems concerning the requirements for good oxygen separation membrane materials as follows.

(1) Polymers with higher regular structures which are needed for higher α values have lower processability because they are insoluble and vice versa.
(2) Polymers with higher α values have lower *P*_O_2__ values and vice versa. (For the detail, see Section 1.4).

### 1.3. Mechanisms of Gas Permselective Membranes (Figure 1)

**Figure 1:**
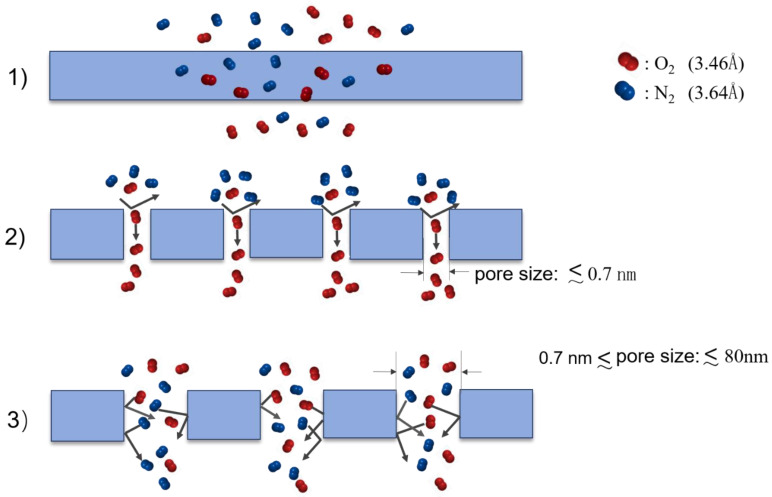
Mechanisms of oxygen selective permeation through membranes. (**1**) Solution-diffusion (SD) mechanism for dense (non-porous) membranes, (**2**) molecular-sieving (MS) mechanism for ultra-microporous membranes, (**3**) Knudsen (Kd) mechanism for microporous and mesoporous membranes.

Because gas molecules are very small particles around 2.6–5.5 Å in kinetic diameter, membranes for gas separation should be dense (non-porous) or microporous and not have any defects whose sizes are more than several Å. Figure 1 summarizes three well-known permselective mechanisms useful for oxygen and nitrogen separation [1,2,3,4,5,6,7,8]. The details of the three are described below.

#### 1.3.1. Solution-Diffusion (SD) Mechanism for Dense (Non-Porous) Membranes

Dense (non-porous) membranes have no pores but gas molecules can permeate them. A popular and the simplest mechanism for gas permeation through dense membranes known as the solution-diffusion (SD) mechanism involves the following two-step process: (i) dissolution (or sorption) of permeating gas molecules on a membrane surface and then (ii) diffusion of the gas molecules across the membrane. In this simple model, the oxygen permeability coefficient (*P*_O_2__: cc (STP) cm cm^−2^ s^−1^ cmHg^−1^) is the product of the oxygen solubility coefficient (*S*_O_2__: cc(STP) cm^−3^ cmHg^−1^) and the oxygen diffusion coefficient (*D*_O_2__: cm^2^ s^−1^).

*P*_O_2__ = *S*_O_2__ × *D*_O_2__ (1)

The oxygen permselectivity, α (*P*_O_2__/*P*_N_2__) is the product of the oxygen solubility selectivity (α_S_ (=*S*_O_2__/*S*_N_2__)) and the oxygen diffusion selectivity (α_D_ (=*D*_O_2__/*D*_N_2__)).

α = α_S_ × α_D_ (2)

Therefore, oxygen separation of membranes based on the SD mechanism depends on the two factors, that is, dissolution (or sorption) selectivity α_S_ (=*S*_O_2__/*S*_N_2__) and diffusion selectivity α_D_ (=*D*_O_2__/*D*_N_2__). Although, in many of gas pairs, if α_S_ > 1, α_D_ < 1 and vice versa. However, in the case of oxygen/nitrogen, α_S_ > 1 and α_D_ > 1 generally.

#### 1.3.2. Molecular-Sieving (MS) Mechanism for Ultramicroporous and Microporous Membranes

When one of the authors (T.A.) published a review article on macromolecular design for permselective membranes in 1999 [1], the molecular-sieving (MS) mechanism was not popular and most oxygen permselective membranes were discussed on the basis of the SD mechanism described above. Recently, it has been thought that this MS mechanism is important for realizing high performance for oxygen separation membranes. Because the sizes of an oxygen and a nitrogen molecule are 3.46 Å and 3.64 Å, respectively, it is ideally desired that static micropores whose sizes are smaller than around 5 Å are created in polymer membranes. However, for common polymers, since 5 Å is within the range of the thermal motion of their polymer backbones and polymer molecules can flex over this range, it is not easy to create static pores whose sizes are less than 5 Å suitable for oxygen separation based on the MS mechanism. 

The MS separation mechanism for an oxygen/nitrogen mixture may be applied to permeations through membranes containing micropores whose sizes are less than around 5–10 Å (0.5–1 nm) because the pore sizes should be less than 2–3 times of the size of the permeating gas molecule (0.346 nm) The IUPAC defined the terms “ultra-micropores” and “micropores” as small pores with sizes of less than 0.7 nm and 2 nm, respectively. Therefore, for highly oxygen permselective membranes based on the MS mechanism, ultra-micropores are appropriate and micropores are too large.

#### 1.3.3. Knudsen (Kd) Mechanism for Microporous and Mesoporous Membranes

When pore sizes in a membrane are smaller than the mean free path (λ) of a permeant gas, the diffusion of the gas follows the Knudsen (Kd) mechanism, where the permeability coefficient is inversely proportional to the square root of the molecular weight of the gas. In the case of oxygen, its λ value is about 60–100 nm. Therefore, when separating oxygen using the membranes with pores whose sizes are from 5–10 Å (0.5–1 nm) to 60–100 nm follows the Knudsen (Kd) mechanism. Its permeation (diffusion) selectivity is expressed by the following equation.

α (*P*_O_2__/*P*_N_2__) = α_S_ (*S*_O_2__/*S*_N_2__) × α_D_ (*D*_O_2__/*D*_N_2__) (3)

Because α_S_ (*S*_O_2__/*S*_N_2__) = 1, in this case,

α (*P*_O_2__/*P*_N_2__) = α_D_ (*D*_O_2__/*D*_N_2__) = (M _N_2__)^1^/^2^/(M _O_2__)^1^/^2^ = 0.93

Therefore, in the case of oxygen/nitrogen mixtures, nitrogen permeates predominantly through Knudsen (Kd) membranes. In addition, because α_S_ makes no contribution for this kind of membrane, separation by selective dissolution is not useful for the case of oxygen/nitrogen separation.

In conclusion, for oxygen permselective membranes, nonporous or ultramicroporous polymer membranes are suitable, that is, membranes with micropores whose sizes are from 5–10 Å (0.5–1 nm).

#### 1.3.4. Transition Range between the Solution-Diffusion (SD) Mechanism for Dense (Non-Porous) Membranes, the Molecular-Sieving (MS) Mechanism for Ultra-Microporous Membranes, and the Knudsen (Kd) Mechanism for Microporous and Mesoporous Membranes

As described above, in the case of oxygen, permeation through membranes with pores whose sizes are less than 60–100 nm follows the Knudsen (Kd) mechanism, and separation based on the molecular-sieving (MS) mechanism for oxygen/nitrogen mixture requires micropores with sizes less than about 5–10 Å (0.5–1 nm). Baker also reported that the transition between the MS mechanism and the solution-diffusion (SD) mechanism is in the range of 0.5–1 nm [9]. In conclusion, the minimum and maximum sizes of actual pores in polymer membranes which can separate oxygen/nitrogen mixtures by the MS mechanism are around 5 Å and 10 Å, respectively.

### 1.4. Robeson’s and Pinnau’s Upper Bound Lines

#### 1.4.1. Existence of Upper Bound Lines in Selectivity-Permeability Plots

**Figure 2:**
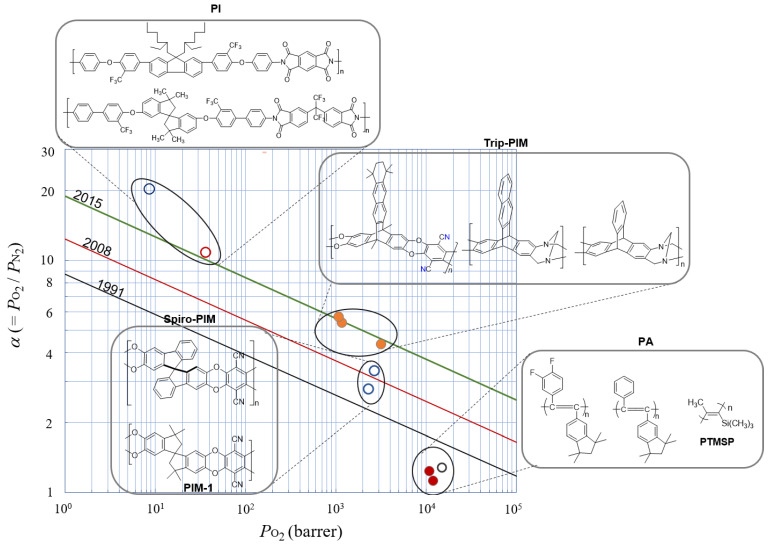
Robeson’s 1991, 2008, and Pinnau’s 2015 upper bound lines for oxygen/nitrogen permeation through polymer membranes with plots for milestone polymers (●: after aging, ○: before aging).

In order to compare oxygen permselectivity, that is, *P*_O_2__ and α (=*P*_O_2__/*P*_N_2__) for various membranes, many researchers used a plot of ln α versus ln *P*_O_2__. In general, trade-off relationships between *P*_O_2__ and α are well-known. Prof. Robeson plotted many experimental values obtained from the literature and drew an upper bound line in 1991 as shown in Figure 2 [10]. This is the original upper bound line. In the 1991 figure, substituted acetylene polymers (PAs) including poly(1-trimethylsilylpropyne) (PTMSP, see Figure 2) and polyimides (PIs) were plotted as very high *P*_O_2__ polymers, and as high α polymers, respectively. The figure also showed that in general, glassy polymers showed better performances than rubbery polymers. The details are described in Section 2.1.

#### 1.4.2. Meaning of the Upper Bound Lines—The Slope and Intercept

Although Robeson’s 1991 upper bound line was drawn from empirical results, Prof. Freeman explained the theoretical meaning of the line in 1999 [11]. He constructed the following equation for the line:

ln α = −**A** ln *P*_O_2__ + **B** (4)

where **A** and **B** indicate the slope of the line and the intercept at ln *P*_O_2__ = 0, respectively. He discussed the meaning of **A** and **B** in the following way.

**A** = (d_N_2__/d_O_2__)^2^ − 1 = (3.64/3.46) ^2^ − 1 = 0.105 (5)

where d_O_2__ and d_N_2__ are kinetic diameters for oxygen and nitrogen, respectively. Since the **A** value is determined by gas pairs as permeants, it has no relationship to the structures of the membrane polymers. Therefore, it cannot be improved by well-organized molecular design based on researchers’ efforts. On the other hand, he proposed that the **B** value is affected by solubility selectivity and the structures of membrane polymers. Therefore, researchers can design new polymers showing good performances by controlling the **B** value. Furthermore, since the solubility selectivity is low in the case of oxygen/nitrogen, precise design of polymers to enhance α_D_ is more important. In order to obtain good performance of polymer membranes, that is, to realize a simultaneous increase in selectivity and permeability, it is effective to increase chain stiffness (main chain rigidity) and interchain spacing (fractional free volume (FFV)).

#### 1.4.3. History of the Upper Bound Lines—From 1991 via 2008 to 2015

When one of the authors (T.A.) published a review article on macromolecular design for permselective membranes in 1999 [1], only Robeson’s 1991 upper bound line was known. Since 1991, the upper line has been moving in the upper-right direction. For example, Robeson’s 2008 upper bound line [12] and Pinnau’s 2015 upper bound line [13] were reported as shown in Figure 2. As Prof. Freeman predicted in 1999 [11], their slopes are the same as shown in Figure 2.

The advent of Robeson’s 2008 upper bound line was caused by the emergence of a new type of polymer called PIM (polymers of intrinsic microporosity) such as PIM-1 as shown in Figure 2. This polymer backbone includes a rigid ladder structure consisting of alternating fused aromatic and aliphatic cycles with spiro-type carbons as kink points. In this review, we call this type of PIMs as Spiro-PIM. The details are described in Section 2.1.4.

Recently, Pinnau’s 2015 upper bound line appeared. The new line was caused by the advent of another type of PIMs. Similar to a Spiro-PIM, these advanced new PIMs contain a rigid ladder structure consisting of alternating fused aromatic and aliphatic cycles. But instead of spiro-type carbons in Spiro-PIMs, they include triptycene groups as kink points (see Figure 2). In this review, we call this type of PIM the Trip-PIM. The details are described in Section 2.2.2.

#### 1.4.4. Aging Problems for Ultrahigh Permeable Polymers

As described above, the ultramicropores which are needed for high performance of oxygen permselective membranes based on the molecular-sieving (MS) mechanism have a similar size to that of the thermal motion of the polymer backbone. In addition, because these pores are mainly present between macromolecules, they are usually unstable, particularly in the case of ultrahigh permeability polymers (*P*_O_2__ > 10^3^) such as PTMSP (the details are described in Section 2.1.2). Therefore, they often showed an aging effect, whereby with increasing time their permeability significantly decreased. *Because we think that the stable values after aging are the real and intrinsic values of polymers, we plotted separately the aged values by solid symbols and transient non-aged values by open symbols in all the figures in this review whenever aged values were reported.*

#### 1.4.5. Advantages and Disadvantages of Polymers as Materials for Oxygen/Nitrogen Permselective Membranes

As described in Section 1.1, soluble polymers are suitable for oxygen/nitrogen permselective membranes because they can be fabricated easily as dense thin membranes. This is the fundamental advantage of polymers. In addition, their chemical structures can be controlled with an accuracy of subnanometer order by organic and polymer synthesis. Therefore, it is possible to control precisely the size of the permeation path of oxygen and nitrogen. However, in the case of linear soluble polymers because permeation paths are mainly present between macromolecules, they are usually unstable (see Section 1.4.4) and it is difficult to control the size.

### 1.5. Purposes of This Review

The main aim of this review is to discuss how to design and obtain new good polymers with both high oxygen permeability (*P*_O_2__) and oxygen permselectivity (α (*P*_O_2__/*P*_N_2__)). For this purpose, we first extensively searched for papers reporting values of α and *P*_O_2__ for polymers with well-defined chemical structures. Then, we selected polymers showing the highest α at each *P*_O_2__ among them. Finally, we plotted them on a (ln α (*P*_O_2__/*P*_N_2__) − ln *P*_O_2__) graph (see Section 2). As described above, if papers reported more than two values before and after aging for the same polymer, we plotted both values as open and solid symbols, respectively. In addition, in order to see the history of recent progress of the research for oxygen permeation polymers, results until 2013 and up to now (until 2020) are discussed separately in Section 2.1 and Section 2.2, respectively. Furthermore, in Section 3, in order to discuss strategies for enhancing *P*_O_2__ and α (*P*_O_2__/*P*_N_2__) simultaneously, we selected such examples from the literature although we found they were quite few. *While we described the performance of only pure polymers in Section 2, we included modified polymers such as composite polymers in Section 3 because of the very rare examples for pure polymers* (For the contents of this review, see Supplementary Materials S1).

Finally, by using these collected data of pure membranes from these top-performing linear soluble polymers and composite membranes including insoluble polymers whose *P*_O_2__ and α (*P*_O_2__/*P*_N_2__) were simultaneously enhanced, we will summarize the relationships between the chemical structures and performances and discuss how to obtain new good polymers with high values for both *P*_O_2__ and α.

## 2. Macromolecular Design for Oxygen/Nitrogen Permselective Membranes—Top-Performing Polymers

### 2.1. Top-Performing Polymers until 2013 as Oxygen/Nitrogen Permselective Membranes

**Chart 1:**
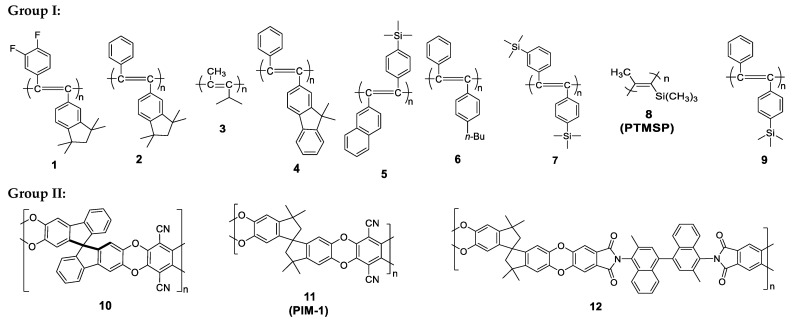

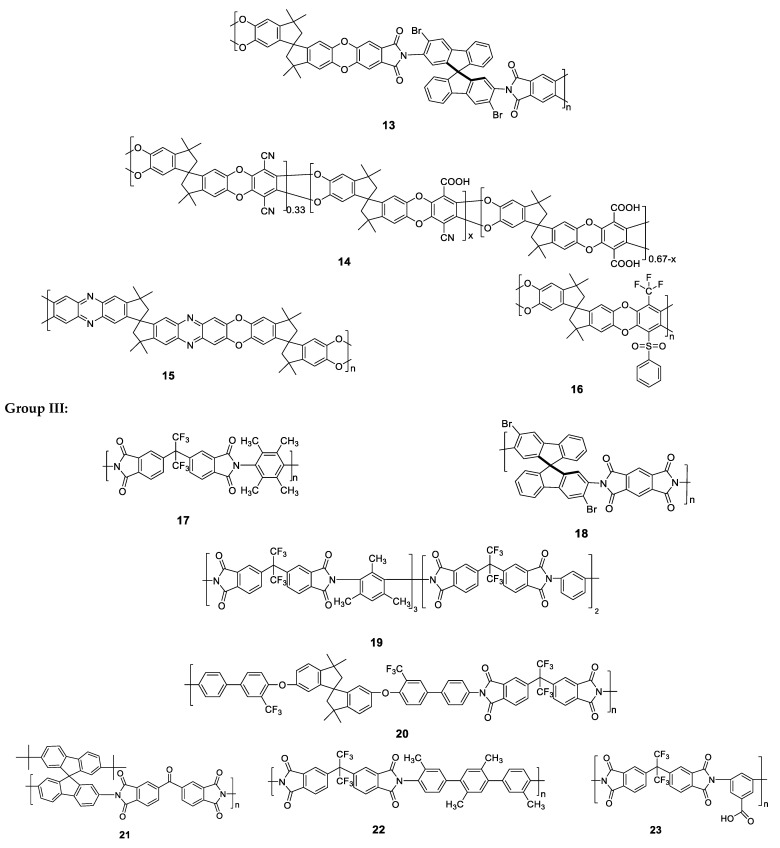
Chemical structures of membrane polymers in Figure 3 (All polymers until 2013).

**Figure 3:**
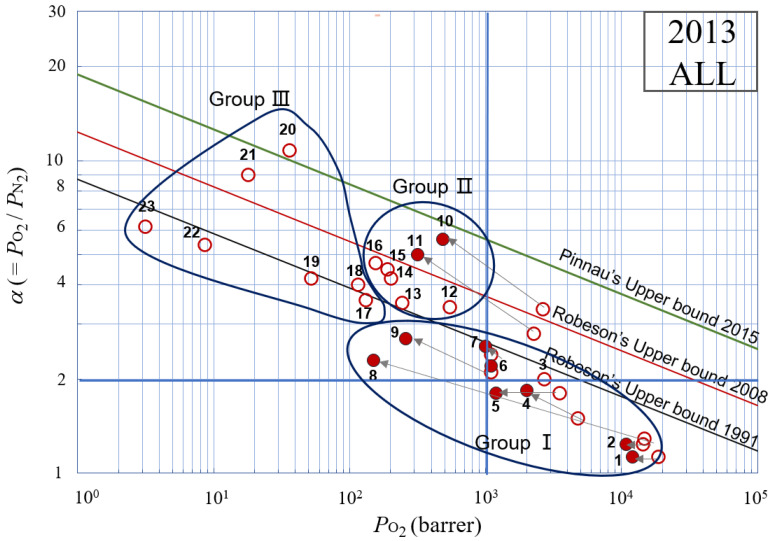
Plots of oxygen permselectivity through polymers, showing the highest α at each *P*_O_2__ reported in the literature until 2013 (●: after aging, ○: before aging). For the data and categorization of the plots, see Table 1, and for the chemical structures of the polymers in the plots, see Chart 1.

Figure 3 shows plots of top-performing polymers showing the highest α at each *P*_O_2__ reported in the literature until 2013. The values and categorization are listed in Table 1 [14,15,16,17,18,19,20,21,22,23,24,25,26,27,28,29,30,31,32,33,34,35]. The chemical structures of the polymers showing the best performances are shown in Chart 1. These polymers can be categorized into the following three groups. Group I: polymers **1**–**9** with the highest *P*_O_2__ and the lowest α below the 1991 upper line, Group II: polymers **10**–**16** with relatively high α and intermediate *P*_O_2__ beyond the 1991 upper line and close to, or on, the 2008 upper line, and Group III: polymers **17**–**23** with the highest α and the lowest *P*_O_2__ close to the 1991 upper line. Typical polymers categorized into Groups I, II, and III are poly (substituted acetylenes) (PAs) such as PTMSP, Spiro-PIMs (polymer of intrinsic microporosity with spiro-carbons), and polyimides (PIs), respectively. The details are described below.

#### 2.1.1. Poly(Substituted Acetylene)s (PAs) (Group I: Polymers **1**–**9** in Chart 1 and Figure 3, and Polymers **24**–**34** in Chart 2 and Figure 4)

**Figure 4:**
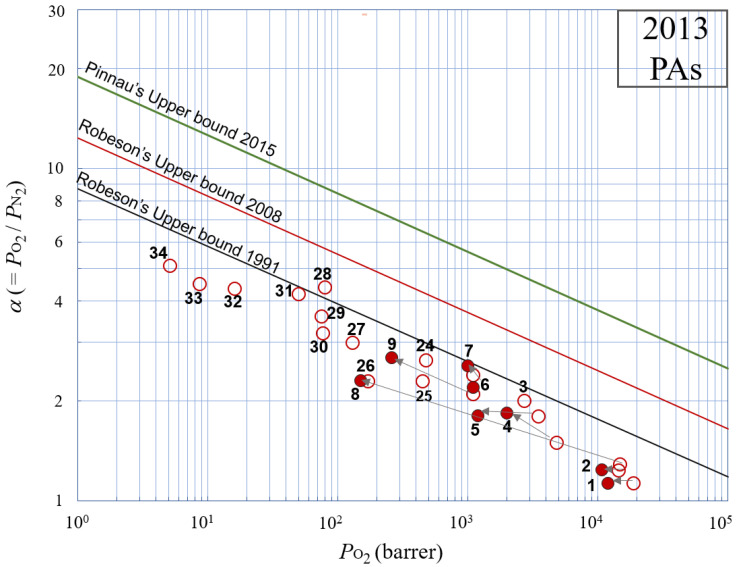
Plots of oxygen permselectivity through poly(substituted acetylenes) (PAs), showing the highest α at each *P*_O_2__ reported in the literature until 2013 (●: after aging, ○: before aging). For the data and categorization of the plots, see Table 2, and for the chemical structures of the polymers in the plots, see Chart 1 and Chart 2.

In the milestone paper which reported Robeson’s 1991 upper bound line [10], it was described that glassy polymers generally show better performances than rubbery polymers. For example, poly (substituted acetylene)s (PAs) are typical glassy polymers with very high glass transition temperatures (Tg) because of their very rigid chemical structures created by their conjugated backbones with bulky and crowded side groups. In fact, PAs reported until 2013 show relatively good performance in oxygen permselectivity as shown in Figure 4 and Table 2 [36,37,38,39,40,41,42,43]. They are plotted at very close to or on Robeson’s 1991 upper bound line but do not exceed the 2008 line until 2013.

Among the best 20 PAs, polymers **7** and **28** show the best and second-best performances, as shown in Figure 4. Polymer **7** lying on the 1991 upper bound line shows ultrahigh *P*_O_2__ around 10^3^ with relatively high α [20]. This polymer has two trimethylsilyl and two phenyl groups. Polymers **9**, **24**, and **26** show lower *P*_O_2__ with similar α values compared with polymer **7**. All these three polymers have one or two trimethylsilyl and two phenyl groups. Among these four PA type polymers, the polymer **7** from the monomer with the greatest number of trimethylsilyl and phenyl groups is the best. These bulky groups may create large amounts of micropores and the high mobility of the trimethylsilyl groups may maintain a high *P*_O_2__. Polymer **28** show the best performance among the best 20 PAs [39]. Only this polymer includes polar atoms, sulfurs. The other nineteen polymers consist of only non-polar atoms, i.e., carbon, silicon, and hydrogen which do not induce polarity in the molecule. Therefore, it is thought the polar structure might enhance the α_S_.

#### 2.1.2. Poly(1-Trimethylsilylpropyne) (PTMSP) (an Ultrapermeable Polymer) (Polymer **8** in Figure 3 and Figure 4)

**Chart 2:**
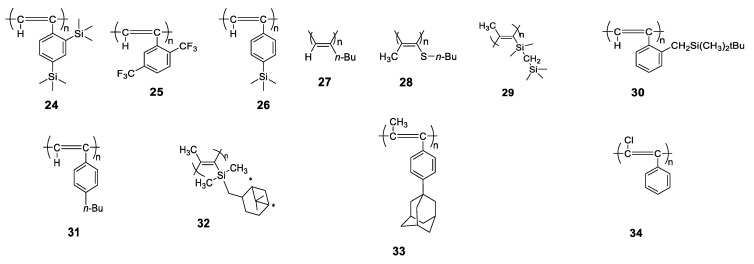
Chemical structures of the membrane polymers in Figure 4 (PAs until 2013). For **1**–**9**, see Chart 1.

Among the PAs in Figure 4 (**1**–**9** in Chart 1 and **24**–**34** in Chart 2), only a few PAs (**1**–**7**) show very high *P*_O_2__ values of more than 10^3^. The highest value is more than 10^4^. Such polymers are called ultrapermeable polymers [44]. They are all poly(disubstituted acetylene)s (DPA). Poly(1-trimethylsilylpropyne) (PTMSP, polymer **8** in Chart 1) [45,46] reported by Prof. Masuda in 1983 is most meritorious among DPAs. The high *P*_O_2__ value (10^4^) was much higher than that (10^2^) for poly(dimethylsiloxane) (PDMS) which had been the highest value before his discovery. After this discovery, a great number of chemists, physicists, and materials scientists started new research projects concerning PTMSP in order to clarify the reason for the extremely high *P*_O_2__ value and to try to apply it to practical use. As a result, it was found that the extremely high *P*_O_2__ was caused by an unusually high fractional free volume (FFV, around 25–30%), which is also sometimes called ultrahigh FFV, or a large amount of very large micropores. The density is also the lowest (around 0.75). Therefore, the polymer is also called a super-glassy polymer. The sizes of the unusually large micropores in PTMSP have been reported in many papers as follows: 0.5–1.0 nm by Bakers [9], 0.9–1.2 nm by Robeson [12], 1.12 nm by Freeman [47], 1.24 nm by Lee [48], 1.1–1.5 nm by Pinnau [49], 0.45–0.55 nm by Yampolskii [50]. Judging from these reports, the sizes of the pores in PTMSP may be around 0.5–1.5 nm.

As described above, in order to separate oxygen (3.46 Å) and nitrogen (3.64 Å) based on the molecular sieving mechanism, pore sizes should be less than 1.0–0.5 nm. Since the sizes of the pores in PTMSP may be around 0.5–1.5 nm as described above, PTMSP is partially suitable for oxygen/nitrogen separation membranes on the basis of the MS mechanism. In fact, it was reported that the permselectivity α (*P*_O_2__/*P*_N_2__) of PTMSP was mainly due to the diffusion selectivity, α_D_ (*D*_O_2__/*D*_N_2__) (=1.4) and no dissolution selectivity, α_S_ (*S*_O_2__/*S*_N_2__) (=1.1) was detected [9,51].

Although the *P*_O_2__ value of PTMSP is very high, the α value is extremely low, around 1.3. The pores in PTMSP whose sizes are around 0.5–1.5 nm are categorized both as micropores (<2.0 nm), useful for high *P*_O_2__, and ultramicropores (<0.7 nm), useful for high α, but PTMSP contains mainly micropores and only a small amount of ultramicropores. Therefore, the *P*_O_2__ is very high and the α is very low. Another possible reason for the extremely low α value is the effect of partial permeation based on the Knudsen (Kd) mechanism. If the permeation was solely based on the Kd mechanism, no dissolution selectivity should be found. Therefore, its α should be α_D_ = 0.93. If the contribution of possible dissolution selectivity values is considered, the α values are 1.5–2.1 [12]. Since it was reported that the oxygen permeation behavior through PTMSP is within the transition range from SD mechanism to Kd mechanism [12], this possible reason may be reasonable. 

A search of papers for *P*_O_2__ values for PTMSPs results in scattered values from 10^4^ to 10^2^. This is unusual. Because a *P*_O_2__ value generally and inherently is one of physical properties of a usual polymer, the *P*_O_2__ should depend on the chemical structure of the polymer. Therefore, a polymer has its own specific *P*_O_2__ value. However, in general, the same polymers do not always produce the same homogeneous dense membranes because their high-order structures such as crystallinity, and their membrane structures, such as an asymmetric structure, are not the same in each case. In the case of PTMSP membranes that have a variety of *P*_O_2__ values, they can have quite different pore sizes. One of the reasons why the reported values of the pore sizes for PTMSPs were scattered as described above is that the sizes depend on the membrane preparation method. This variation of the pore sizes suggests instability of the micropores. In fact, strong aging effects were reported for the *P*_O_2__ values. The pore sizes must have been also decreasing with time. This may be another reason for the scattered data for the pore sizes. In fact, the very high *P*_O_2__ decreased significantly from 10^4^ to 10^2^ over time. The change is shown in Figure 3 and Figure 4 (●: after aging, ○: before aging). 

Although PTMSP and PDMS similarly show very high *P*_O_2__ values, the physical properties of PTMSP and PDMS are completely different and therefore their permeations are based on different permeation mechanisms. While PTMSP is a very rigid super-glassy polymer (whose Tg is thought to be more than 200 °C) with the lowest density, PDMS is a very flexible polymer with the lowest Tg = −123 °C. PTMSP has static micropores but PDMS has no micropores. As a result, permeation through PTMSP mainly follows the MS mechanism and permeation through PDMS follows the SD mechanism, although their performances are similar.

#### 2.1.3. Poly(Diphenylacetylene)s (PDPAs) (Polymers **1**, **2**, **4**–**7**, and **9** in Chart 1)

In Figure 4, there are some poly(substituted acetylene)s showing ultrapermeability, i.e., *P*_O_2__ of more than 10^3^ even after aging (polymers **1**–**7**), although PTMSP shows ultrapermeability only before aging. Most of them are poly(diphenylacetylene)s (PDPAs) (polymers **1**–**7**, except for **3**). PDPAs have a more rigid backbone than PAs in general because they have bulky substituted phenyl groups at every carbon of the main chain. Although they show very high *P*_O_2__ even after aging similar to non-aged PTMSP, their α values tend to be low and further from the upper line compared with other PAs. This may be caused by the permeation mechanism including not only the SD mechanism but also the Kd mechanism. In particular, the two PDPAs with indan groups (polymers **1** and **2**) show extremely high *P*_O_2__ of more than 10^4^ even after aging, similar to non-aged PTMSP. There are almost no aging effects on their performances, a situation quite different from PTMSP, as shown in Figure 3 (●: after aging, ○: before aging).

#### 2.1.4. Spiro-PIMs (Polymers of Intrinsic Microporosity with Spirocarbons) (Group II: Polymers **10**–**16** in Figure 3 and Chart 1)

In the plots of the best polymers until 2013 (Figure 3), although the plots of the polymers showing high *P*_O_2__ of more than 10^3^ (PDPAs) are below the 1991 upper bound line, the polymers (**10**–**16**) showing intermediate *P*_O_2__ values from 10^2^ to 10^3^ are located above the 1991 upper bound line. All these polymers are called PIMs (polymers of intrinsic microporosity) and related PIMs. Therefore, the new class of polymer contributed significantly to the appearance of the new improved 2008 upper line.

(1) Advent of the first PIM (PIM-1, **11** in Chart 1 and Figure 3)

The first PIM with an excellent *P*_O_2__ and α (**11** in Chart 1 and Figure 3) was reported in the non-aged state by Budd and McKeown in 2005 [29]. Although network amorphous polymers like carbon molecular sieve membranes (CMSMs) contain a large fractional free volume (FFV), they were insoluble and had no processability for their self-supporting membranes. Therefore, they designed a soluble non-network amorphous polymer containing a large FFV, called PIM-1 (no.11 in Figure 3). The new polymer is a linear macromolecule consisting of a very rigid fused rings backbone connected by spiro-carbons at contorted positions (In this review, this type of PIM is called Spiro-PIM). Because this is a linear (ladder) polymer, it is soluble and processable. In addition, the rigid ladder (fused rings) backbone with the kink points (spiro-carbons) can create relatively stable large micropores. As a result, it shows good performance beyond the 1991 upper line. The α for non-aged PIM-1 (**11**○ in Chart 1 and Figure 3) [25] is much higher than that for non-aged PTMSP (**8** ○ in Chart 1 and Figure 3) [14], although the *P*_O_2__ for non-aged PIM-1 is much lower than that of PTMSP. The plot for PIM-1 lies above the 2008 upper bound after aging, although the *P*_O_2__ is less than 10^3^. It was reported that the selectivity of permeation (α = 2.8) mainly depended on that of diffusion (α_D_ = 2.8) and that of dissolution (α_S_ = 1.0) was almost unity (no selectivity) suggesting it follows the MS mechanism [24]. This better α is because the size of pores, determined by nitrogen adsorption, in PIM-1 was much smaller (around 6.5 Å) than that (around 9 Å) in PTMSP [13].

(2) Improved Spiro-PIM (polymers **10** and **15** in Chart 1)

In addition, to enhance the rigidity of the main chain of PIM-1, a bulky group (fluorene) was introduced around the spirocarbons (**10**) or the fused rings backbone was extended (**15**). Compared with PIM-1 (**11** in Chart 1 and Figure 3) both the *P*_O_2__ and α increased for PIM **10**. This is because the introduction of the additional fused benzene rings restricted motion around the spiro-center of the backbone. The selectivity of permeation (α = 3.4) mainly depended on that of diffusion (α_D_ = 3.4) and that of dissolution (α_S_ = 1.0) was almost unity (no selectivity) suggesting it follows the MS mechanism [24]. In the case of no.15 PIM, both the α and *P*_O_2__ decreased compared with no. 11. The lower ratio of the kink points may enhance the stacking of the backbones to reduce their micropores or FFV.

The two Spiro-PIMs (**10** and **11** in Chart 1) described above showed strong aging effects as shown in Figure 3 (● and ○), similar to PTMSP (**8**). Therefore, several different α and *P*_O_2__ values were reported by different researchers. Both of the two PIMs lie above the 2008 upper bound after aging, although *P*_O_2__ values are less than 10^3^.

(3) Spiro-PIMs with side groups (modified Spiro-PIMs) (**14** and **16** in Chart 1)

Side groups were introduced to Spiro-PIMs (**14** and **16**). Compared with PIM-1 (**11**), both α and *P*_O_2__ decreased. The polar groups may enhance intermolecular interactions to reduce their micropores or FFV.

(4) Spiro-PIM-PI (**12** and **13** in Chart 1)

The other related PIMs that are plotted above the 1991 upper bound line are new PIMs which include imido-structures (Spiro-PIM-PI, **12** and **13**). However, compared with PIM-1 (**11**), their performances are not superior. Introducing the simple single bonds of C–N into the PIMs’ ladder backbones may reduce the rigidity which is effective for good performance. However, compared with usual polyimides describe below, they have higher *P*_O_2__ values.

#### 2.1.5. Polyimides (PIs) (Group III: **17**–**23** in Chart 1 and Figure 3, and **35**–**43** in Chart 3 and Figure 5)

In Figure 3 for all the best polymers until 2013, polymers **17**–**23** show high α values and low *P*_O_2__ values. They are all polyimides (PIs). Among them, polymers **20** and **21** show the top two highest α values. They contain contorted backbones. Therefore, they are also categorized as Spiro-PIM-PI as mentioned above.

**Figure 5:**
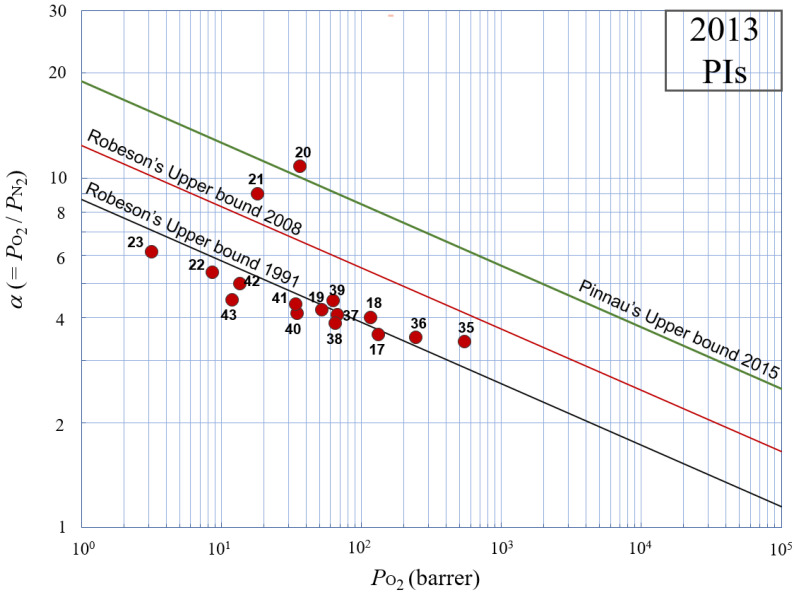
Plots of oxygen permselectivity through polyimides (PIs), showing the highest α at each *P*_O_2__ reported in the literature until 2013. For the data and categorization of the plots, see Table 3, and for the chemical structures of the polymers in the plots, see Chart 1 and Chart 3.

**Chart 3:**
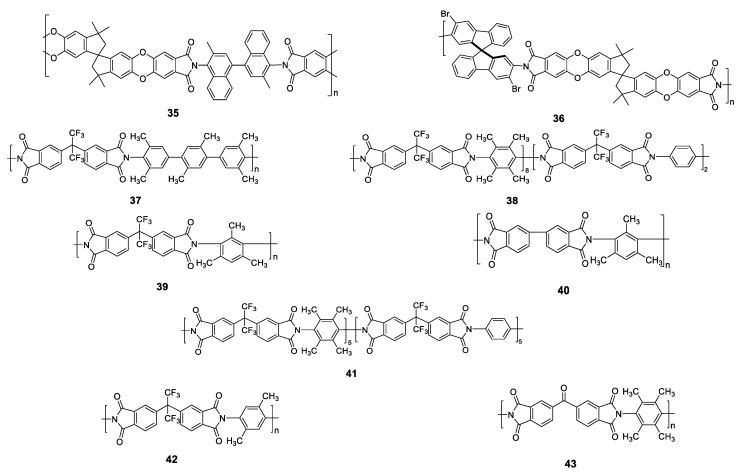
Chemical structures of the membrane polymers in Figure 5 (PIs until 2013). For **17**–**23**, see Chart 1.

Figure 5 shows plots of the best PIs until 2013. Table 3 lists the detailed data for the polymers in Figure 5 [52,53,54]. They are amorphous and have very high Tg values, that is, they are glassy polymers. Conventional PIs are known as insoluble polymers and, therefore, they have no direct processability and their membranes are prepared from their soluble precursor polymers called poly(amic acid)s [55]. However, by introducing bis(trifluoromethyl)methylene (-(CF_3_)_2_C-) to the main chain of PIs, they became soluble and therefore direct fabrication to their membranes became possible. The bulky groups inhibit efficient packing of the polymer chains resulting in higher permeability. In fact, almost all the PIs showing the best performances here in Figure 5 include the fluorine-containing group (-(CF_3_)_2_C-), except for polymers **40** and **43** and PIM-PIs (**35**, **36**, **18**, **20**, and **21**)) (Chart 1 and Chart 3). The PIs can be classified into two groups. One group contains a lot of methyl groups (polymers **17**, **19**, **22**, **37**–**44**) and the other have contorted spiro carbons (polymers **18**, **20**, **21**, **35**, and **36**; PIM-PI, the details are described above). The PIs with many methyl groups (**39**, **19**, **41**, **22**) had relatively high densities around 1.3–1.4 and low FFVs of about 10–20%. However, they had relatively large spaces between macromolecules, because their *d*-spacing values from XRD were 0.46–0.57 nm [56] and their pore sizes by PALS [48] were 0.6–0.7 nm. In other words, these PIs may contain ultramicropores (<0.7 nm). It was reported that their selectivities of permeation (α) through these PIs mainly depended on those of diffusion (α_D_) and those of dissolution (α_S_) were almost unity (<1.2) [32,52]. Therefore, since the permeation may involve the MS mechanism, relatively high α values are shown. However, since they have no large micropores (0.7–1.0 nm), unlike PTMSP, their *P*_O_2__ values were low.

### 2.2. Top-Performing Polymers in 2020 as Oxygen/Nitrogen Permselective Membranes

**Figure 6:**
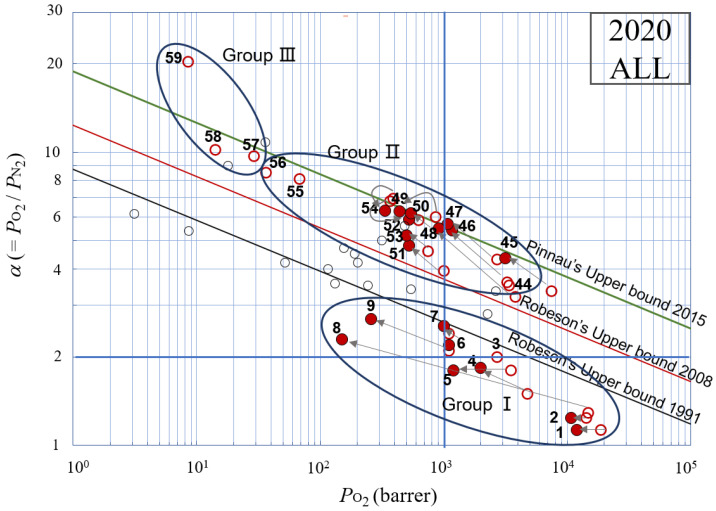
Plots of oxygen permselectivity through polymers, showing the highest α at each *P*_O_2__ reported in the literature in 2020 (●: after aging, ○: before aging). For the data and categorization of the plots, see Table 4, and for the chemical structures of the polymers in the plots, see Chart 1 and Chart 4. The small open circles indicate data in Figure 3. [Note] Please compare this figure with Figure 3. You can recognize a big advance between 2013–2020 and also no advance of polymers in Group I.

**Chart 4:**
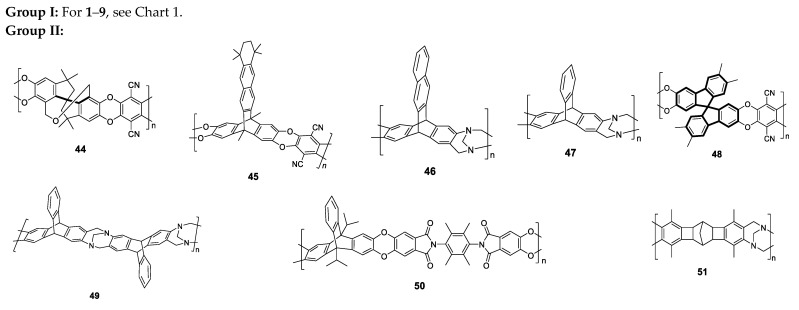

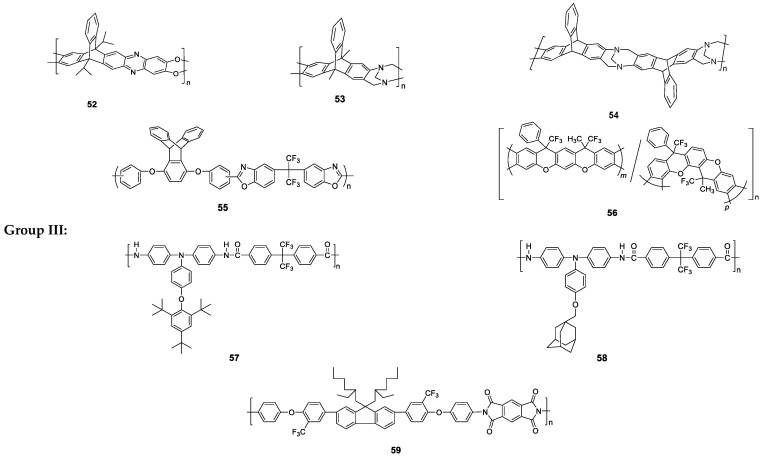
Chemical structures of the membrane polymers in Figure 6 (all polymers in 2020).

After we had reviewed the data until 2013 and summarized them as described in 2.1, we then thoroughly searched for reports published from 2014 to the present and renewed the plots for top-performing polymers from the previous plots until 2013 (Figure 3) to the latest plots (Figure 6) of polymers showing the highest α at each *P*_O_2__ reported until 2020. The values and categorization are listed in Table 4 [57,58,59,60,61,62,63,64,65,66,67,68,69]. The chemical structures of these polymers showing the best performances are shown in Chart 4. It is very surprising that many high-performance polymers which are beyond the 2008 upper bound line appeared in this period of 2014–the present. It is very clear that the progress is very rapid by comparing the changes for six years in 2014–2020 in Figure 6 with those for the 22 years 1991–2013 in Figure 3 (The plots in Figure 3 are also shown by open small circles with thin lines in Figure 6).

The polymers can be categorized into the following three groups. Group I: polymers **1**–**9** (Chart 1) with the highest *P*_O_2__ and the lowest α below the 1991 upper bound line, Group II: polymers **44**–**56** (Chart 4) with high α and high *P*_O_2__ beyond the 2008 upper bound line and close to, or on, the 2015 upper bound line, and Group III: polymers **57**–**59** (Chart 4) with the highest α and the lowest *P*_O_2__ beyond the 2008 upper line and close to, or beyond, the 2015 upper bound line. Typical polymers categorized into Groups I, II, and III are poly (disubstituted acetylenes) such as poly (1-trimethylsilylpropyne) (PTMSP) and poly(diphenylacetylene)(PDPA), ladder polymers with side arylene groups without spirocarbons (Trip-PIM), and polyamides (PAm) or polyimide (PI) with bulky pendant groups, respectively. The details are described below.

#### 2.2.1. Poly(diphenylacetylene)s (PDPAs) and Modified PAs (Group I; **1**–**9** in Figure 6 and Chart 1)

(1) Poly(diphenylacetylene)s (PDPAs)

In the plots of this region in Figure 6 (polymers **1**–**9** in Chart 1), the situation is similar to that until 2013 (Figure 3, polymers **1**–**9**). Therefore, no noticeable improvements in this region have occurred in the period since 2014 to the present (for the discussion, see Section 2.1.3).

**Figure 7:**
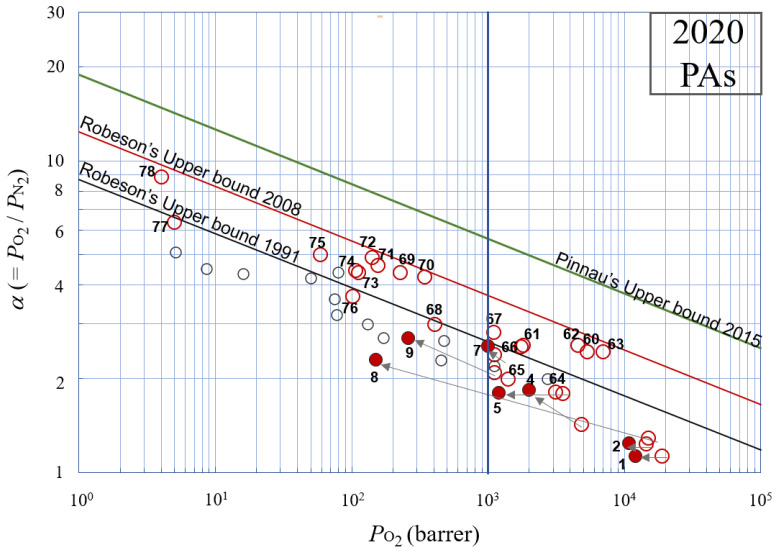
Plots of oxygen permselectivity through polyacetylenes (PAs), showing the highest α at each *P*_O_2__ reported in the literature in 2020 (●: after aging, ○: before aging). For the data and categorization of the plots, see Table 5, and for the chemical structures of the polymers in the plots, see Chart 1 and Chart 5.

**Chart 5:**
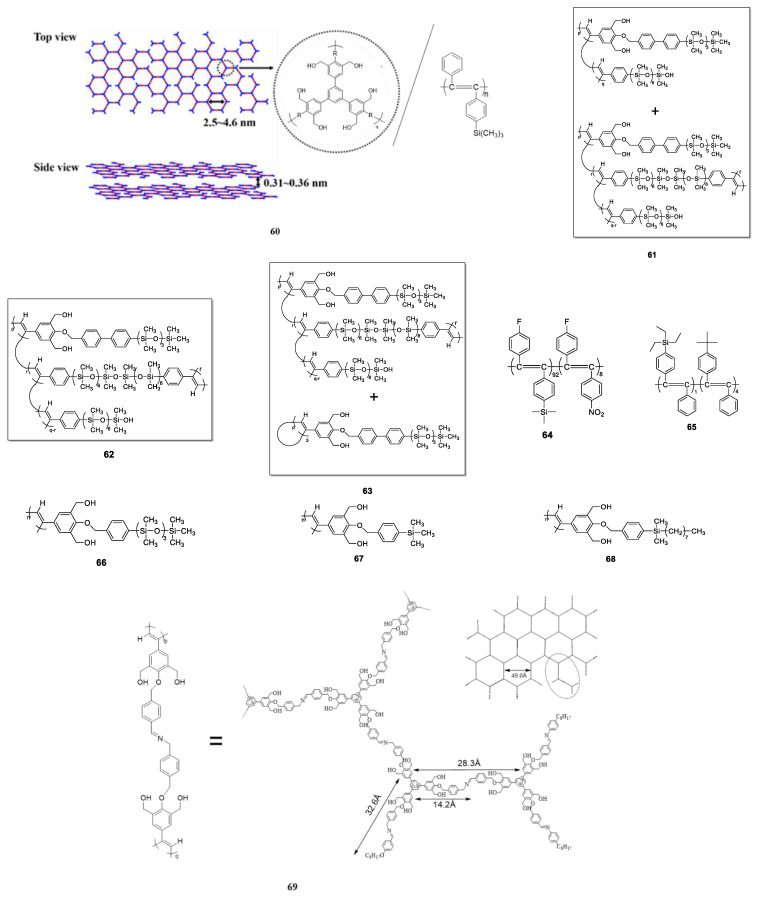

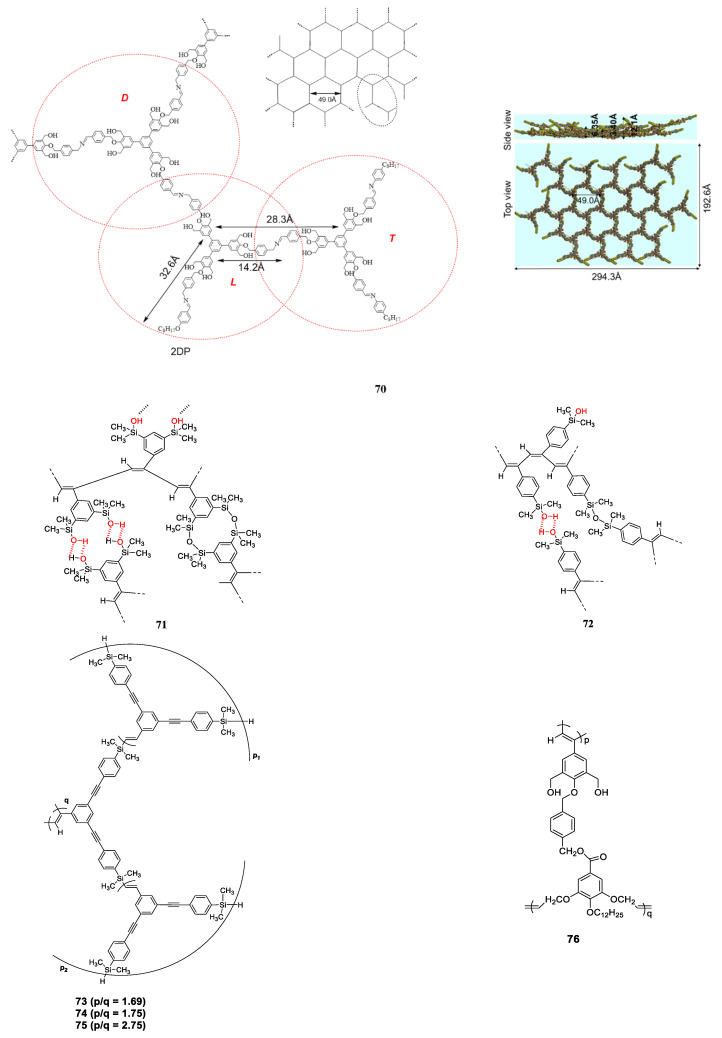

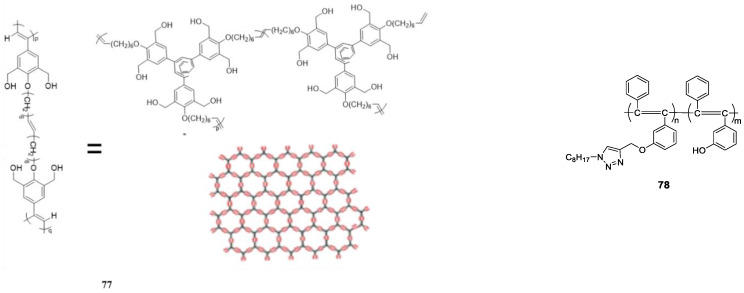
Chemical structures of the membrane polymers in Figure 7 (PAs in 2020). For **1**–**9**, see Chart 1.

(2) Modified PAs (**60**–**78** in Figure 7 and Chart 5;)

Figure 7 shows plots of all the PAs having good performance reported from 2014 to the present. The detailed data are listed in Table 5 [70,71,72,73,74,75,76,77,78,79]. Although until 2013, all the plots for PAs were below the 1991 upper bound line as shown in Figure 4 (the plots in Figure 4 are also shown by open small circles with thin lines in Figure 7), some of the plots for PAs in 2020 are located above the 1991 upper bound line and close to the 2008 upper bound line. Such developments have been realized using several methods by the author’s group. The methods are categorized into the following three main groups. Category A: changing to more rigid cis-cis main chains in PAs than cis-trans PAs together with introducing soft linear siloxane side groups (c-c PAs and their derivatives; polymers **61**–**63**, **66**–**68**, and **76**, **77** in Chart 5); Category B: introducing hyperbranched or soft crosslinked structures (network structures) into cis-trans PAs (c-t PAs and their derivatives; **71**–**75** in Chart 5); Category C: membranes containing network two-dimensional (2D) polymers derived from cis-cis PAs (c-t PAs and their SCAT (highly selective photocyclic aromatization) derivatives; **60** and **70** in Chart 5). In category A, since cis-cisoid polyphenylacetylenes (c-c PAs) are more rigid than c-t PAs and similarly to PDPAs, some c-c PAs show better performance than c-t PAs. In particular, a combination of the rigid backbone and soft and short oligosiloxane grafts are suitable (**66**) [74]. Introduction of soft crosslinking and modification by SCAT reaction of these types of polymer enhanced their performances (**61**–**63**) [71]. In category B, introducing hyperbranched (**73**–**75**) [77] or soft network (**71** and **72**) [76] structures to c-t PAs enhanced their permselectivities. In category C, by embedding two-dimensional (2D) polymers created by SCAT reaction of c-c PAs to membranes, good performances were realized. The 2DP **70** [75] was synthesized by interfacial polycondensation at the solid–solid interface and 2DP **60** membrane [70] was prepared by blending with polymer **9**. In summary, it was found that a rigid backbone based on c-c PAs, soft short side chains and network structures including hyperbranched and 2DP structures were effective for enhancement of α values. Rigidification of PA’s main chain enhanced α and *P*_O_2__ simultaneously (**66**) [74]. However, network structures (**69**, **71**, **72**, and **77**) including a multi-stranded structure (**76**) [78] caused a decrease of *P*_O_2__ compared with the precursor liner polymers, although α increased. Only in the cases of hyperbranched (**73**–**75**) [77] and 2DP structures (**60** [70] and **70** [75]), was *P*_O_2__ also maintained.

#### 2.2.2. Ladder Polymers with Side Arylene Groups without Spirocarbons and Other Ladder Polymers (Group II; Polymers **44**–**56** in Figure 6 and Chart 4)

(1) Ladder polymers with side arylene groups without spirocarbons (Trip-PIMs)

As shown in Figure 6 for all the polymers until 2020, great developments are observed in the region between 10^2^ and 10^3^ (ultrapermeable region) of *P*_O_2__ compared with all the polymers until 2013 shown in Figure 3. They (**44**–**56**) are located above the 2008 upper bound line and in particular some of the polymers (**45**–**50**, **52**, and **54**) are located far above the 2008 upper bound line. Pinnau’s 2015 upper bound line [13] was newly drawn because the three polymers (**47**, **50**, and **52**) appeared in 2014. Most of them (except for **44** and **51**) are a new type of PIM having rigid ladder backbones similar to Spiro-PIM. However, the new PIMs contain no spirocarbons in their main chains and instead they contain triptycene (Trip) groups as kink points in their backbones. Therefore, in this review they are called Trip-PIM. By contrast with Spiro-PIMs described in Section 2.1.4, the backbones of Trip-PIMs have totally ladder structures. 

Although the three polymers reported in 2014 (**47**, **50**, and **52**) made a significant contribution to the new 2015 upper bound line [13], their *P*_O_2__ values were much less than 10^3^ (ultrapermeable region) and lower than that for PTMSP (**8** in Chart 1) (as described above, non-aged PTMSP has a large amount of micropores around 13 Å which is effective for the very high *P*_O_2__.) These excellent performances of Trip-PIMs were caused by the effective size distribution of ultra-micropores (<7 Å) and micropores (<20 Å). For example, in the case of Trip-PIM **52**, the sizes of ultramicropores [27] are smaller (around 5.3 Å) than those of Spiro-PIM (PIM-1) (around 6–7 Å) [13,27]. In addition, both the PIMs have similar amounts of a larger size of micropores (around 13 Å) useful for high permeability. Therefore, this Trip-PIM shows much better performance than the Spiro-PIM(PIM-1). Table 6 summarizes data of α (*P*_O_2__/*P*_N_2__), α_S_ (*S*_O_2__/*S*_N_2__), and α_D_ (*D*_O_2__/*D*_N_2__) values for some of Trip-PIMs (**46**, **47**, and **53**) plotted in Figure 6. For these polymers, the high α values depend only on α_D_ and their α_S_ values were around unity.

Some substituent effects on the performances of Trip-PIMs were examined. The size of arylene groups as the side groups and the size or shape of alkyl groups at the bridge heads were changed. When the phenylenes (**47** in Figure 6) [59] are changed to larger naphthylenes (**46**) [58], the *P*_O_2__ increases and α decreases. These α rely almost exclusively on α_D_ as shown in Table 6. The spaces may be enlarged by enhancing the size of the substituents. The bulkier substituents may create micropores suitable for *P*_O_2__ enhancement. On the other hand, when the alkyl groups at the bridge heads are changed from hydrogen groups (=no substituent, **47**) ([59]) to bigger methyl groups (**53**), ref. [64] both *P*_O_2__ and α decrease at the same time. The small substituents may fill the original micropores. When the alkyl groups at the bridge heads were changed from *n*-propyl groups (*P*_O_2__ = 101, α = 5.7 [63] to *iso*-propyl groups (**52**), ref. [63] both *P*_O_2__ and α increased. These α values were almost exclusively on α_D_ as shown in Table 6. The rigidity may be increased by enhancing the bulkiness of the substituents and, therefore, ultramicropores suitable for α enhancement may form. In fact, the pore sizes were found to become narrower based on NLDFT analysis. 

Among Trip-PIMs **47**, **49**, and **54** (Figure 6 and Chart 4) which are isomers to one another, and are all composed of triptycenes and Tröger’s bases, polymer **54** shows the best α. The main chain of polymer **54** is relatively straight, while the others are not straight. Therefore, polymer **54** may create the smallest micropores among the three. Actually, polymer **54** has the highest density (0.931), lowest FFV (0.349), and smallest pore sizes (5.7 Å) [60].

Recently among Trip-PIMs, a new polymer (**45**) was reported to have extremely high *P*_O_2__ of more than 10^3^, i.e., ultrapermeability while maintaining their excellent α values (>4). This is the best performance up to the present [44]. This Trip-PIM has a totally complete ladder backbone having no Tröger’s base units. In addition to the fact that Trip-PIMs have generally smaller ultramicropores than Spiro-PIMs as described above, this Trip-PIM also has a much higher population of larger micropores (7–10 Å) effective for enhancing *P*_O_2__ than a Spiro-PIM. Therefore, polymer **45** shows the best performance having both ultrahigh *P*_O_2__ and high α values. In fact, the FFV is very large (0.309), similar to PTMSP (Table 6).

(2) PIs with Trip

Polymer **50** showing one of the best performances is a polyimide containing Trip units. Several Trip-PIM-PIs were synthesized from different diamines [53]. Among them, polymer **50** showed the best performance. Because it contains tetramethylphenylene units, the rigidity of the C–N bonds may be the highest.

(3) Other ladder polymers

Two novel ladder polymers with no Trip groups and spiro carbons in their backbones (**51** and **56**) were reported as some of the best performance polymers. While Trip-PIMs are totally straight ladder structures with bulky side groups (1,2-phenylene groups), these new ladder polymers have no bulky side groups like a Trip. Since their ladder backbones are not straight instead, they may have micropores. However, their performances are inferior to Trip-PIMs. Similar to the Trip-PIMs containing Tröger’s base units (**47**, **49**, and **54**) mentioned above, the ladder polymer **51** containing Tröger’s base units showed a similar tendency. The pure isomer polymer had a higher α and lower *P*_O_2__.

(4) Modified Spiro-PIMs

The polymers **44** [57] and **48** [23] are modified Spiro-PIMs. The former polymer has a more rigid backbone made by introducing additional cyclic structure around the relatively flexible spirocarbons. In other words, this is a locked PIM-1 (**11** in Chart 1). As a result, both *P*_O_2__ and α values are increased simultaneously. This may be caused by increasing the rigidity producing a longer interchain distance. The latter BenzoSpiro-PIM (**48**) with four methyl groups shows higher *P*_O_2__ and lower α than the original BenzoSpiro-PIM (**10** in Chart 1 and Figure 3) without substituents.

**Figure 8:**
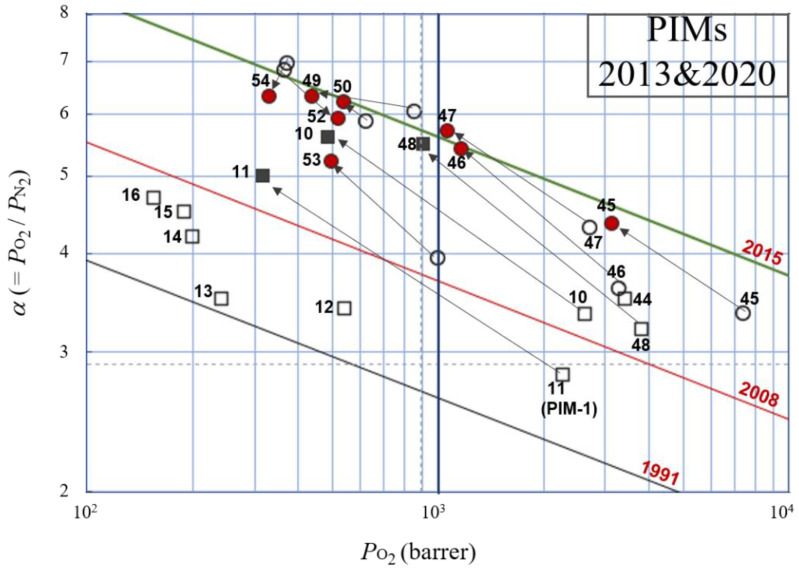
Plots of oxygen permselectivity through polymers of intrinsic porosities (PIMs), showing the highest α at each *P*_O_2__ reported in the literature until 2013 and 2020. For the numbers see Chart 1 (2013) and Chart 4 (2020). ●○: Trip-PIM, ■□: Spiro-PIM, ●■: aged values, □○: none-aged values.

Figure 8 summarizes the performance and progress of all the PIMs, that is, Spiro-PIMs and Trip-PIMs. It shows clearly that both *P*_O_2__ and α values are improved by a change from Spiro- to Trip-PIMs. In addition, they show a large decrease in *P*_O_2__ by aging. As a consequence, most aged PIMs are not ultrapermeable, except for polymers **45**–**47**. However, plots for most of Trip-PIMs are located on the 2015 upper bound line at the aged state.

#### 2.2.3. PI or Polyamides (PAms) with Bulky Side Groups (Group III, **57**–**59** in Figure 6 and Chart 4)

As described in 2.1.5, the polyimide (PI) (**20** reported in 2010 in Chart 1 and Figure 3 until 2013) shows an unusually high α value (10.8). In this case, kink points were introduced to a PI by spirocarbon structures in a similar way to Spiro-PIMs. In Figure 6 in 2020, some condensation polymers of not only PI (**59** in Chart 4 and Figure 6) [69] but also polyamides (PAms) (**57** and **58** in Chart 4 and Figure 6) [67,68] show extremely high α values from 10 to 20. The PI has spiro-carbons (fluorene) and bulky alkyl side groups and the PAms have bulky side groups (tris(*tert*-butyl)phenoxy and adamantyl groups). As a result, their *d*-spacing values (5.9–6.2 Å) [67,68,69] determined by XRD were larger than those (4.6–5.7 Å) [55] for conventional PIs. 

Table 6 summarizes data of α (*P*_O_2__/*P*_N_2__), α_S_ (*S*_O_2__/*S*_N_2__), and α_D_ (*D*_O_2__/*D*_N_2__) values from the literature for some of the polymers plotted in Figure 6. For these condensation polymers (**57**–**59** in Chart 4 and Table 6), the extremely high α values depend not only on α_D_ but also on α_S_ This is much different from Trip-PIMs whose α (*P*_O_2__/*P*_N_2__) values depend only on α_D_ values because the α_S_ values are almost unity. Therefore, these extremely high α values could be realized by high α_S_ values in addition to high α_D_ values. It suggests the mechanism of this permeation partially involves the SD mechanism.

**Figure 9:**
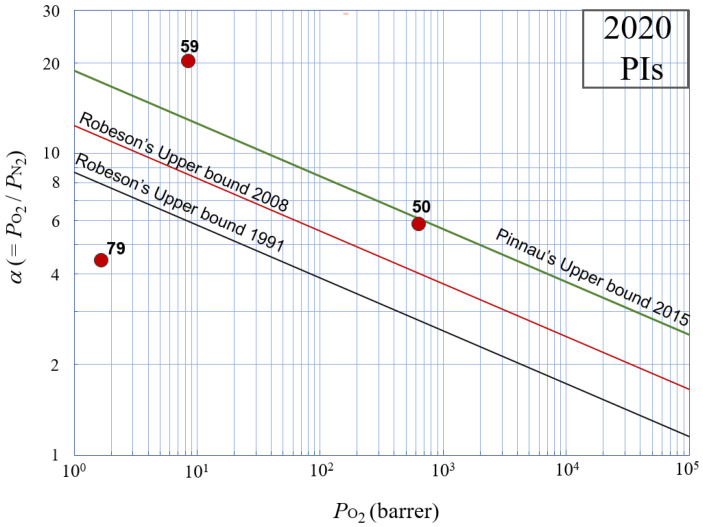
Plots of oxygen permselectivity through polyimides (PI), showing the highest α at each *P*_O_2__ reported in the literature in 2020. For the data and categorization of the plots, see Table 7, and for the chemical structures of the polymers in the plots, see Chart 6.

Figure 9 shows the present situation for PIs. In the plots for PIs reported until the present, the situation is similar to those in Figure 5 and Table 7 [80] and only a few noticeable improvements were reported during the period (**50** and **59**). They possess kink points or bulky groups in their backbones.

### 2.3. Tendency of Change in Oxygen Permeation Performances by Changing Chemical Structures of Polymers 

As described above as shown in Figure 3, Figure 4, Figure 5, Figure 6, Figure 7, Figure 8 and Figure 9, it was found that almost all the changes in the oxygen permeation performances by changing the polymer chemical structures are from lower-right positions to upper-left positions and vice versa in these α − *P*_O_2__ plots. They moved parallel to the upper bound lines in the figures. In other words, whenever their permeability increased, their permselectivity decreased and vice versa. In the case of aging causing physical changes, they also moved parallel to the upper bound lines (for example, Figure 8). However, we found some welcome exceptions in which the permeability and permselectivity increased at the same time as described in this section (Section 2). Here we summarize the exceptions as follows.

- Changing their backbone structures:

(1) Linear polymers to ladder polymers (ex. **8**● (PTMSP) to **51**● (a ladder) in Chart 4 and Figure 6 and Figure 8● (aged PTMSP) to **10**●and **11**● (aged PIMs) in Chart 1 and Figure 3).
(2) Ladder polymers to ladder polymers with kink points (Trip-PIMs) (ex. **51**● to **47**● in Chart 4 and Figure 6)
(3) Liner polymers (PIs) to linear polymers with kink points (PIM-PIs) (ex. **22** and **23** to **20** and **21** in Chart 1 and Figure 3)
(4) Spiro-PIMs to Trip-PIMs (ex. **14**■ (Chart 1) to **47**● (Chart 4) in Figure 8)
(5) Spiro-PIM (non-aged) (**11**○in Chart 1 and Figure 8) to locked Spiro-PIM (**44** in Chart 4 and Figure 8)
(6) c-t PA to c-c PA (ex. **66** in Chart 5 and Figure 7)

Therefore, as can be seen in (1)–(6), it can be concluded that rigidification of main chains is very effective method for producing such simultaneous improvements. In addition to this, introducing contorted sites in the main chains is important.

(7) Linear polymers to 2D network polymers (**60** and **70** in Chart 5 and Figure 7)

Crosslinking generally forms 3D network structures and often decreases *P*_O_2__ values, although α values increase effectively (**77** in Figure 7). On the other hand, 2D network structures could enhance both *P*_O_2__ and α values (**60** and **70** in Chart 5 and Figure 7). Also, soft-crosslinking is suitable for avoidance of loss of *P*_O_2__ values (**71** and **72** in Chart 5 and Figure 7).

II. Changing their side groups:

(8) *n*-Propyl Trip-PIM to *iso*-propyl Trip-PIM (**52**●in Chart 4 and Figure 8)
(9) Spiro-PIM (**11**■ in Chart 1 and Figure 8) to benzospiro-PIM (**10**■ in Chart 1 and Figure 8)
(10) Introduction of hyperbranched structures (**73**–**75** in Chart 5 and Figure 7)
(11) PI to PI with bulky pendant groups (**22** and **23** in Chart 1 and Figure 3 to **59** in Chart 4 and Figure 6)
(12) Methyl Trip-PIM (**53**● in Chart 4 and Figure 8) to unsubstituted Trip-PIM (**47**● in Chart 4 and Figure 8)

Therefore, as can be seen in (8)–(11), it can be concluded that bulkier side groups may create spaces suitable for simultaneously enhancing both *P*_O_2__ and α values. But sometimes small and flexible substituents can unfavorably occupy spaces between macromolecules. In such cases, the performances were lowered. In fact, such a case was observed in (12). The influence of side groups in polymers with high FFV on their performances is not simple.

## 3. Simultaneous Improvements of Selectivity and Permeability of Polymer Membranes

In Section 2, performances for pure membranes from mainly only pure polymers whose chemical structures are clear are plotted in Figure 3, Figure 4, Figure 5, Figure 6, Figure 7, Figure 8 and Figure 9 except for Figure 7 because the main aim of this review is to discuss the relationships between macromolecular structures and their performances. However, the performances usually changed in upper-left directions or lower-right directions, that is, the changes were often parallel to the upper bound lines when chemical structures were changed. In other words, when *P*_O_2__ values increased, their α values decreased and vice versa. Although improvement of both *P*_O_2__ and α values at the same time is very desirable, observations of such improvements were very rare, especially in pure polymer membranes. Therefore, in order to discuss how both *P*_O_2__ and α values can be increased simultaneously, in this section we will discuss membranes made from physically-modified polymers whose structures are not clear and also composite membranes made from polymers containing porous additives.

### 3.1. Polymer Modification by Thermal Polymer Reactions 

#### 3.1.1. Thermally Rearranged Polymers (TRs) 

Thermal rearrangement is one of the effective methods for improving both *P*_O_2__ and α values simultaneously [81]. Typically, polyimides (PIs) with hydroxyl pendant groups can be thermally converted to poly(benzoxazole)(PBO) by heating around 400 °C (For example, **55** in Chart 4 and Figure 6) [65]. In general, thermally rearranged polymers (TRs) have much higher *P*_O_2__ values than the original polymers with similar or slightly higher α values. The TRs have both large (8–11 Å) and small (3–5 Å) pores, although the precursor hydroxyl PIs have only intermediate sized (6–7 Å) pores (these sizes were determined by the PALS method). The smaller pores (ultramicropores) are important for the high α values and the larger pores (micropores) are important for the high *P*_O_2__ values [48]. 

#### 3.1.2. Carbon Molecular Sieve Membranes (CMSM)

**Figure 10:**
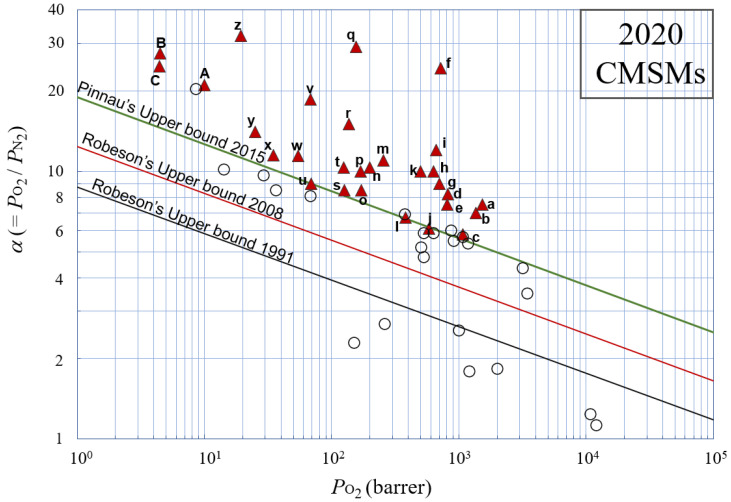
Plots of oxygen permselectivity through carbon molecular sieve membranes (CMSM), showing the highest α at each *P*_O_2__ reported in the literature in 2020. For the data and categorization of the precursor polymers of the plots, see Table 8, and for the chemical structures of the precursor polymers of the plots, see Chart 7.

**Chart 7:**
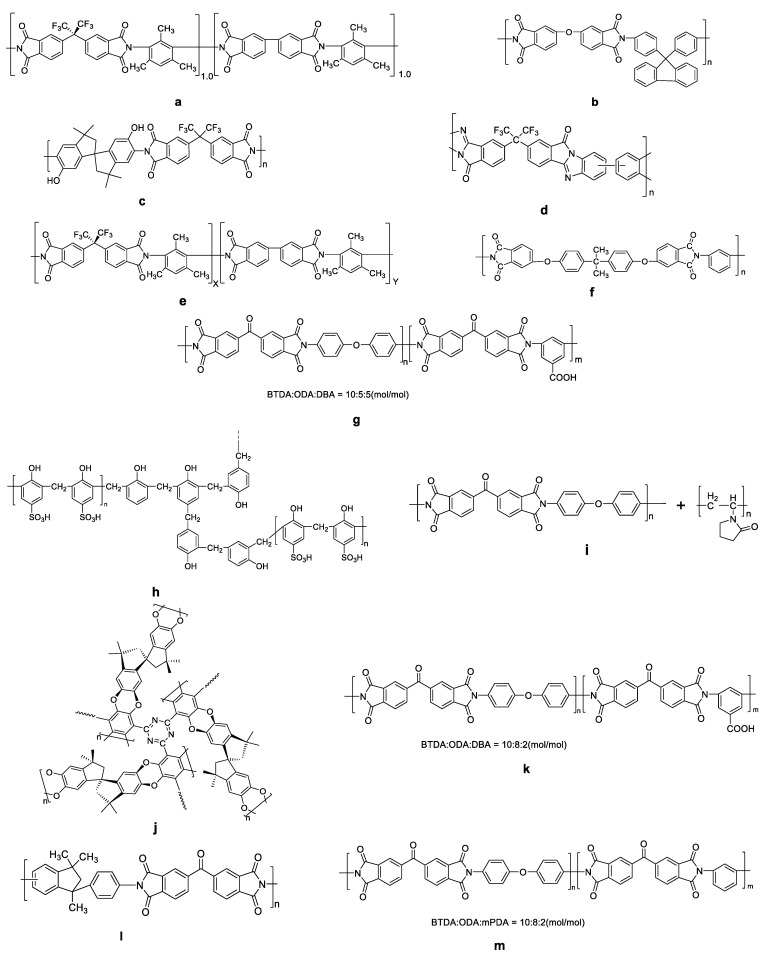

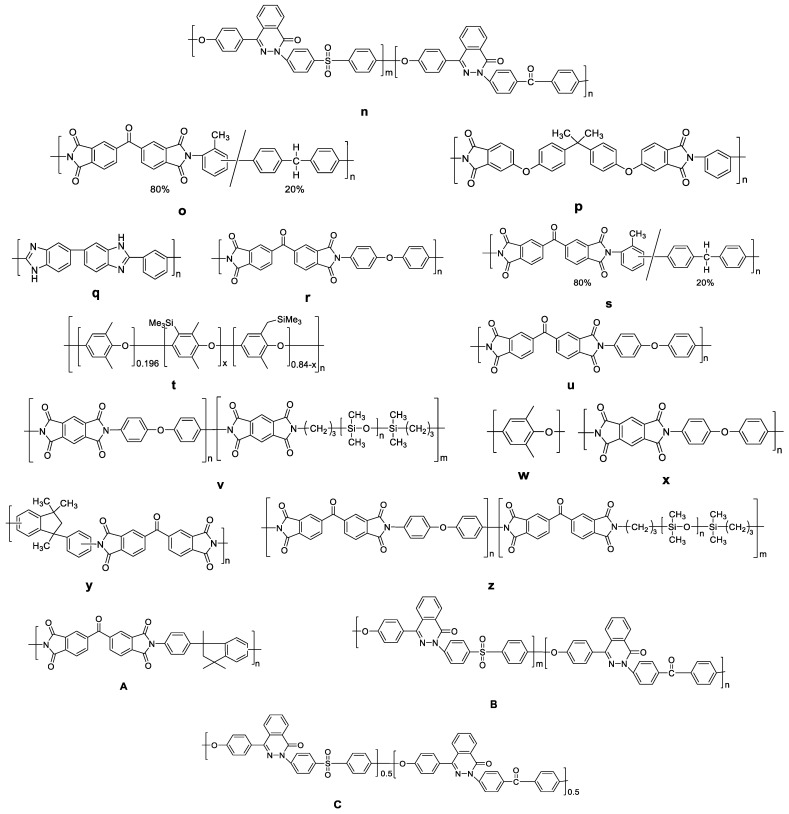
Chemical structures of the precursor polymers of CMSMs in Figure 10 (CMSMs in 2020).

Organic polymers are used as precursors of carbon molecular sieve membranes (CMSM). The precursor polymers are polyimides (PIs), polysulfones (PSfs), poly(phenylene oxide)s (PPOs) and so on. Figure 10 shows plots of oxygen permeation behaviors for CMSMs. Table 8 lists the detailed data [82,83,84,85,86,87,88,89,90,91,92,93,94,95,96,97,98,99,100,101,102,103]. There are many plots for CMSMs above the 2015 upper bound line for organic polymers. Many CMSMs show the best performance among all the membranes and much better performance than organic polymers, although most of them do not show ultrapermeablity. CMSMs are generally prepared by pyrolysis by heating of the precursor polymers to more than about 550 °C. Although fabrication of CMSMs that are thin and large in area is generally more difficult and expensive than that of organic polymers, their performance is much better than those of organic polymers. In particular, CMSMs **f**, **g**, **z**, **A**, and **B** have very high α values of more than 20. Although the original *P*_O_2__ values of the precursor polymers seem to affect those of the resulting CMSMs, these α values for the CMSMs were not influenced by those of the precursor polymers (Chart 7). Since CMSMs **z**, **A**, **B**, and **C** showing high α values of more than 20 were prepared at higher temperatures (at 800 or 900 °C) than usual CMCMs, the temperature seems to be more important for α values than the chemical structures of the precursor polymers.

Since CMSMs are insoluble and amorphous materials which are composed of solely carbon atoms without any organic functional groups, it is difficult to discuss the relationships between macromolecular structures and their performance. Instead, this section focuses on pore sizes in CMSMs.

**Figure 11:**
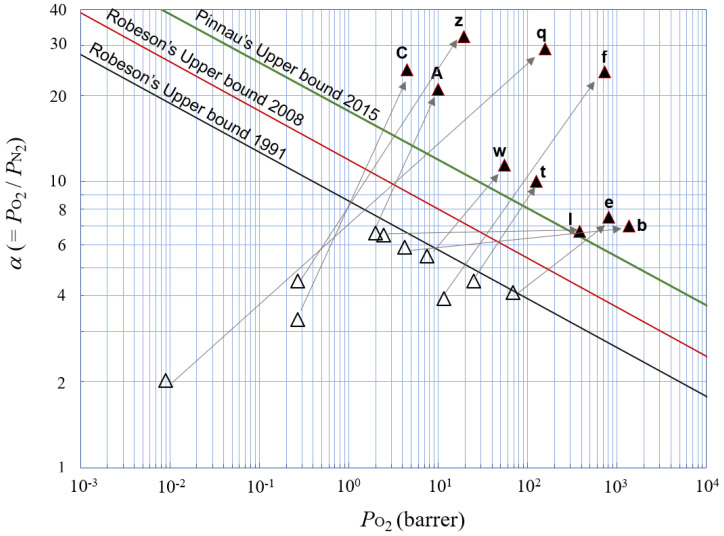
Change in the α and *P*_O_2__ from the precursor polymers to carbon molecular sieve membranes (CMSMs). For the codes, data and categorization of the precursor polymers of the plots, see Chart 7.

Figure 11 shows change of performances from the precursor polymers to the resulting CMSMs showing excellent performance. They all change in the direction of upper-right significantly, that is, simultaneous enhancements of *P*_O_2__ and α values are observed. In particular, the changes of the performance from their precursors to the CMSMs **f**, **g**, **z**, **A**, and **C** are very large. These desirable changes come about because the CMSMs may have both ultramicropores contributing high α and micropores contributing high *P*_O_2__, although the precursor polymers may have only intermediate-sized pores [101]. These high α values were caused by their high α_D_ values. Their α_S_ values were almost at unity because CMSMs had no functional groups which could selectively interact with gas molecules. CMSM **A** (in Figure 9 and Figure 10) has performance (*P*_O_2__ = 10, α = 21) similar to a polyimide (*P*_O_2__ = 8.5, α = 20; Figure 6 and Chart 4, **59**) with bulky pendant groups showing the highest α value among all the polymers described in Section 2.2.3. However, the α value of CMSM depends only on the α_D_ value (=17), but the α value of the polyimide depended both on the α_D_ (=7) and α_S_ values (=3). (Table 6) Their separation mechanisms are different. Generally, both ultramicropores and micropores were created simultaneously by pyrolysis of precursor polymers such as polyimides. When the pyrolysis temperature was high, the size of the ultramicropores became smaller. Therefore, CMSMs prepared at higher temperatures showed higher α values and lower *P*_O_2__ values. In other words, by increasing the pyrolysis temperature, α values increase and *P*_O_2__ values decrease [101].

### 3.2. Polymer Membrane Modification by Blending with Microporous Additives—Mixed Matrix Membranes (MMMs)

As described in Section 3.1, in order to enhance *P*_O_2__ and α values simultaneously for a membrane, both ultramicropores and micropores should be created in the membrane. Preparation of CMSMs by pyrolysis of polymers is a very suitable method. However, control of the membrane preparation process and the pore sizes are still challenging. As another methodology for preparation of membranes with good mechanical strength and micropores whose sizes are well-controlled, mixed matrix membranes (MMMs) have been recently developed [104,105,106,107]. Typically, an MMM consists of a soluble amorphous organic polymer which shows good membrane-forming ability as a matrix and insoluble crystalline inorganic porous compounds as an additive or a filler which has no membrane-forming ability and well-defined micro- or ultramicropores. In this section, we will describe examples of the simultaneous increase in *P*_O_2__ and α values found in the studies on MMMs. Because such examples are very rare, especially for separation of the mixture of O_2_ and N_2_ whose size is 3.46 Å and 3.64 Å, respectively (their size difference is 0.18 Å), here we will include results from studies on separation performances for the other mixtures. For example, the mixture of CO_2_ and CH_4_ whose size is 3.30 Å and 3.80 Å, respectively (their size difference is 0.50 Å) and the mixture of CO_2_ and N_2_ whose size is 3.30 Å and 3.64 Å, respectively (their size difference is 0.34 Å), are described.

#### 3.2.1. Required Conditions for Simultaneous Improvements of Selectivity and Permeability by the MMM Method

In order to use effectively both the roles of matrix polymers and porous additives in MMMs, it is necessary that the two components do not interact negatively with each other and their boundaries produce no defects. To avoid forming such defects, compatibility of polymers with additives in MMMs is important.

#### 3.2.2. Types of Microporous Additives

(1) Zeolites [104]

In combinations of zeolite 4 AA (pore size: 3.8 Å) (25 wt%) [108] and polysulfones, and zeolite 5 A(pore size: 4.8 Å) (50 wt%) and poly(ether sulfone) [109], simultaneous improvements were reported from *P*_O_2__ = 1.3 barrer and α = 5.9 to *P*_O_2__ = 1.8 barrer and α = 7.7, and from *P*_O_2__ = 0.47 barrer and α = 5.8 to *P*_O_2__ = 0.70 barrer and α = 7.4, respectively. On the other hand, in the combinations of zeolite 4A and poly(vinyl acetate), poly(ether imide), or polyimide, α increased but *P*_O_2__ decreased. The decrease may be caused by blocking of their micropores in the zeolite by the polymers. The selection of the matrix polymers is important.

(2) Metal organic frameworks (MOF) [105]

Because metal organic frameworks (MOF) include organic ligands, the compatibility of MOFs with organic polymers should be higher than that of purely inorganic zeolites. In addition, to reduce the formation of defects between polymers and MOF fillers, introduction of functional groups such as amino or acid groups was effective. For example, MMMs of sulfonated MOFs with sulfonated polyimides showed higher *P*_CO_2__ and *P*_CO_2__/*P*_CH_4__ than those of non-modified polyimides [110]. MMMs of an amine-functionalized MOF and a polyimide also showed higher *P*_CO_2__ and *P*_CO_2__/*P*_CH_4__ than the polyimide itself [111,112]. Similarly, in the combination of a polyimide-grafted MOF and a polyimide, a simultaneous increase of *P*_CO_2__ and *P*_CO_2__/*P*_CH_4__ values was observed [113].

(3) Microporous organic cages (POC) [107]

Because microporous organic cages (POC) are purely organic compounds, the compatibility of POCs with organic polymers should be much higher than that of MOFs described above. For example, in an MMM consisting of an imine POC and PIM-1, by addition of the POC, *P*_CO_2__ and *P*_CO_2__/*P*_N_2__ simultaneously increased [114]. In the case of the mixture of O_2_ and N_2_, the addition of a *β*-cyclodextrin (the pore size is 6.0–6.4 Å) as a POC to a polyimide resulted in only a slight increase in α with a slight decrease in *P*_O_2__ [115].

#### 3.2.3. Sizes (Nanocrystals), Shapes (Nanosheets), and Assembled Structures of Microporous Additives

The sizes of additives in MMMs showed large effects on performance. For example, when nanocrystals of a MOF were added to a polyimide, both the *P*C_2_H_2_ and *P*C_2_H_2_/*P*C_2_H_4_ values were enhanced, although when the bulk MOF was used the *P*C_2_H_2_ increased but *P*C_2_H_2_/*P*C_2_H_4_ decreased [116]. The nanosize crystals may avoid the production of defects. The shape of additives also had great effects. In the case of a nanosheet of a MOF accumulated on a macroporous support, by suppressing stackings of the nanosheets, *P*_H_2__ and *P*_H_2__/*P*_CO_2__ increased simultaneously [117]. An MMM based on polybenzimidazole using a nanosheet of a MOF showed simultaneous increases in *P*_H_2__ and *P*_H_2__/*P*_CO_2__. On the other hand, when nanocrystals were used instead, *P*_H_2__/*P*_CO_2__ increased but *P*_H_2__ decreased [118]. Similarly, only a nanosheet of a MOF was effective for enhancing *P*_CO_2__/*P*_CH_4__ (although *P*_CO_2__ decreased), while nanocrystals of the MOF were not effective [119]. 

## 4. Concluding Remarks

### 4.1. High Permeable Linear Polymers, PTMSP and PIMs

The ultrahigh *P*_O_2__ (>10^4^) of very rigid polymers consisting of alternating double bonds with bulky substituents, that is, PTMSP (**8** in Chart 1 and Figure 3), was reported by Prof. Masuda in 1983 [45] and it was confirmed that it contains micropores and its permeation is based on the MS mechanism. Then a new ladder polymer with spirocarbons as contorted points, that is, PIM-1 (**11** in Chart 1 and Figure 3), was developed by Prof. Budd and McKeown in 2005 [29]. With the advent of this new polymer, the original Robeson’s 1991 upper bound [10] was revised in 2008 [12]. Finally, Prof. Pinnau updated Robeson’s 2008 upper bound in 2015 after several Trip-PIMs emerged (**47**, **50**, and **52** in Chart 4 and Figure 6) in 2014 [59,61,63]. Recently, the best PIM having higher ultrapermeability and a very high α was reported by Prof. McKeown in 2017 [44]. Furthermore, it was reported that a new polyimide (polymer **25** in Figure 6) having bulky substituents showed extremely high α (=20.3) by Prof. Banerjee in 2016 [69]. The rate of progress during 2014–2020 was much faster than that during 1991–2013, although the former term (7 years) is much shorter than the latter (23 years). 

The separations of these high-performance polymers are based on mainly α_D_, particularly for polymers with high *P*_O_2__ values such as PTMSP and PIMs. (Although excellent research on oxygen permselective membranes based on α_S_ using metal complexes as oxygen carriers has been carried out by Prof. Nishide, this review does not describe this work because it is outside its scope [120]) In other words, it depends on the MS mechanism. Therefore, the size distribution of their micro- and ultramicropores is decisive and the control of their sizes is important. However, since the micropores are created as spaces between macromolecules, the control of the size is not easy and in addition their stability is low. In fact, their *P*_O_2__ values decreased significantly with time (see Figure 8). This is a big problem for these polymers in terms of practical applications.

### 4.2. High Permselective Inorganic Membranes, CMSM and Graphene

When the chemical structure of a polymer was changed, their performances often changed in the direction of the upper-left or lower-right in the α − *P*_O_2__ graphs. In other words, their direction of change is parallel to the upper bound lines. The most desirable change of improvements of their performances is a movement in the direction of the upper-right, i.e., a simultaneous increase in *P*_O_2__ and α. However, such examples were very rare, as described above. Treatment with physical factors such as heat [121] and light [122] resulted in an increase in α with a decrease in *P*_O_2__ and vice versa. However, we noticed extreme changes in the direction of the upper-right for the preparation of CMSMs from their precursor polymers by heating at 550–800 °C (Figure 11). Although heat treatment of polymer membranes usually affects just high-ordered structures, in this case of formation of CMSMs, changes in chemical structures happened, that is, carbonization with evolution of organic vapers and gases. However, the resulting CMSMs were insoluble and amorphous and, therefore, it is not easy to prepare wide thin membranes of CMSMs like polymer membranes. In addition, it is difficult to know their precise chemical structures from NMR and XRD. The details of the chemical structures are not known but it was thought they may contain graphene sheets. Although graphene macromolecules themselves have no internal pores and, therefore, no filtration functions based on the MS mechanism, the 2D shape seems to be suitable for enhancing performances. (Although some graphenes were applied to gas separation membranes [123,124], their selective permeation occurred during permeation through spaces between graphene sheets or internal defects in graphene sheets.) 

### 4.3. Promising Membranes from Regularly Networked Organic Polymers, 2DPs

Some researchers reported networked 2DPs or nanosheets which are similar to graphene but have micropores whose sizes are similar to those of gas molecules and tried to apply them for gas separation membranes. An example of synthetic 2DPs is a two-dimensional covalent framework (2DCOF) [125]. However, since COFs are crystalline and insoluble, reports of successful applications to oxygen separation membranes were almost zero. Instead we tried to use some kinds of networked polymers and the related polymers made from linear polymers described in Section 2.2.1(2). By adding networked structures, α values often increased but *P*_O_2__ decreased. In the case of networks prepared by short flexible crosslinking, the decrease of *P*_O_2__ was suppressed [76]. In particular, when networked 2DPs was introduced in situ into linear polymers in the membrane state, both α and *P*_O_2__ increased [75]. Although application of 2D materials to separation membranes was reported recently, there have been very few studies on gas separation. In particular, to the best of our knowledge, there have been no reports on the application of 2DP to oxygen permselective membranes [126]. Judging from all these reports, membranes from organic polymers containing organic network 2DPs inside the membranes seem to be a very promising avenue to pursue.

## Figures and Tables

**Chart 6:**
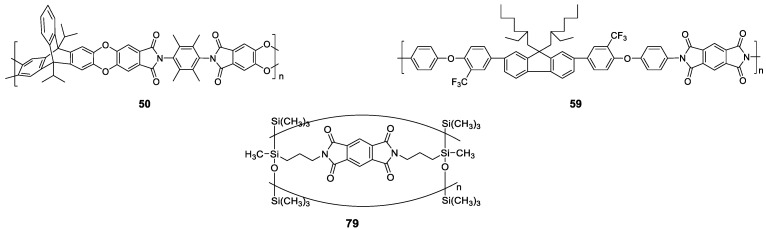
Chemical structures of the membrane polymers in Figure 9 (PIs in 2020).

**Table 1:** Categorization and detailed data for membrane polymers in Figure 3 (All polymers until 2013).

Code	Polymer Type	*P*_O_2__ (barrer)	α (=*P*_O_2__/*P*_N_2__)	Aging Time (days)	Thickness (μm)	Feed Gas	Ref. (year)
Group I							
**1**	**PA**	12,000	1.13	90	80–120	pure	[14] (2008)
		(18,700)	(1.13)				
**2**	**PA**	10,800	1.24	60	-	pure	[15] (2007)
		(14,400)	(1.24)				
**3**	**PA**	(2700)	(2.00)	-	20–40	pure	[16] (1996)
**4**	**PA**	2000	1.84	90	80	pure	[17] (2009)
		(4800)	(1.50)				
**5**	**PA**	1200	1.80	90	-	pure	[18] (2006)
		(3500)	(1.80)				
**6**	**PA**	1100	2.20	-	-	pure	[19] (1994)
**7**	**PA**	1000	2.55	90	30–120	mixed	[20] (2002)
		(1100)	(2.40)				
**8**	**PA** **(PTMSP)**	150	2.30	1460	-	pure	[21] (1993)
		(14,800)	(1.29)				[14] (2008)
**9**	**PA**	260	2.70	26 h	-	-	[22] (1992)
		(1100)	(2.10)				
Group II							
**10**	**Spiro-PIM**	486	5.60	2088	100	pure	[23] (2018)
		(2640)	(3.35)		180	pure	[24] (2012)
**11**	**Spiro-PIM** **(PIM-1)**	317	5.00	1380	128	pure	[25] (2015)
		(2270)	(2.80)		-	pure	[24] (2012)
**12**	**Spiro-PIM-PI**	(545)	(3.40)		96	pure	[26] (2008)
**13**	**Spiro-PIM-PI**	(243)	(3.50)		80–100	pure	[27] (2013)
**14**	**Modified Spiro-PIM**	(201)	(4.20)		70–90	pure	[28] (2009)
**15**	**Spiro-PIM**	(190)	(4.50)		28	pure	[29] (2005)
**16**	**Modified Spiro-PIM**	(156)	(4.70)		50–70	pure	[30] (2008)
Group III							
**17**	**PI**	(132)	(3.57)		50	pure	[31] (2001)
**18**	**Spiro-PIM-PI**	(116)	(4.00)		80–100	pure	[27] (2013)
**19**	**PI**	(52.0)	(4.20)		60	pure	[32] (2013)
**20**	**Spiro-PIM-PI**	(36.1)	(10.8)		85	pure	[33] (2010)
**21**	**Spiro-** **PI**	(18.0)	(9.00)		30–60	pure	[34] (2005)
**22**	**PI**	(8.60)	(5.38)		30–90	pure	[35] (1999)
**23**	**Modified PI**	(3.14)	(6.16)		60	mixed	[32] (2013)

[Note] Data in parentheses are those before aging. For the codes for polymers, see, Chart 1. For the codes for polymer types, see the text.

**Table 2:** Categorization and detail data for the membrane polymers in Figure 4 (PAs until 2013).

Code	*P*_O_2__ (Barrer)	α (=*P*_O_2__/*P*_N_2__)	Thickness (μm)	Feed Gas	Ref. (Year)
**24**	(473)	(2.65)	-	mixed	[36] (1994)
**25**	(450)	(2.30)	30–100	mixed	[37] (1992)
**26**	(171)	(2.30)	-	mixed	[36] (1994)
**27**	(130)	(3.00)	10–100	pure	[38] (1988)
**28**	(79.0)	(4.40)	-	pure	[39] (1990)
**29**	(75.0)	(3.60)	10–100	pure	[38] (1988)
**30**	(77.0)	(3.20)	-	-	[40] (1995)
**31**	(50.0)	(4.20)	-	-	[41] (1998)
**32**	(16.0)	(4.35)	61.9–90.3	mixed	[42] (1996)
**33**	(8.60)	(4.50)	-	-	[43] (1999)
**34**	(5.10)	(5.10)	10–100	pure	[38] (1988)

[Note] For the codes, see Chart 2 and **1**–**9** are the same as those in Table 1. Data in parentheses are ones before aging.

**Table 3:** Categorization and detail data for the membrane polymers in **Figure 5** (PIs until 2013).

Code	*P*_O_2__ (Barrer)	α (=*P*_O_2__/*P*_N_2__)	Thickness (μm)	Feed Gas	Ref. (Year)
**35**	545	3.40	96	pure	[26] (2008)
**36**	243	3.50	80–100	pure	[27] (2013)
**37**	67.0	4.06	30–90	pure	[35] (1999)
**38**	64.8	3.84	-	pure	[52] (2000)
**39**	62.5	4.46	30	mixed	[53] (1999)
**40**	34.7	4.10	-	mixed	[54] (1992)
**41**	33.8	4.37	-	pure	[52] (2000)
**42**	13.4	5.00	-	mixed	[54] (1992)
**43**	11.8	4.48	30	mixed	[53] (1999)

[Note] For the codes, see Chart 3.

**Table 4:** Categorization and detailed data for the membrane polymers in Figure 6 (all polymers in 2020).

Code	Polymer Type	*P*_O_2__ (Barrer)	α (=*P*_O_2__/*P*_N_2__)	Thickness (μm)	Casting Solvent	Feed Gas	Aging Time (Days)	Ref. (Year)
				Group I (see Table 1)				
**Group II**								
**44**	**Spiro-PIM**	(3410)	(3.50)	-	chloroform	pure	-	[57] (2016)
**45**	**Trip-PIM**	3160	4.35	187	-	pure	365	[44] (2017)
		(7470)	(3.35)	(195)				
**46**	**Trip-PIM**	1170	5.40	180	chloroform	pure	166	[58] (2015)
		(3290)	(3.60)					
**47**	**Trip-PIM**	1073	5.70	132	chloroform	pure	100	[59] (2014)
		(2718)	(4.30)					
**48**	**Spiro-PIM**	910	5.50	115	chroloform	pure	1295	[23] (2018)
		(3820)	(3.20)					
**49**	**Trip-PIM**	440	6.29	60	chloroform	pure	150	[60] (2020)
		(864)	(6.01)					
**50**	**Trip-PIM-PI**	542	6.20	77	chroloform	pure	150	[61] (2014)
		(627)	(5.86)					
**51**	**Ladder**	528	4.80	39	chloroform	pure	300	[62] (2020)
		(747)	(4.60)					
**52**	**Trip-PIM**	525	5.90	-	chroloform	pure	15	[44] (2017)
		(368)	(6.80)	-				[63] (2014)
**53**	**Trip-PIM**	500	5.21	-	chloroform	pure	407	[64] (2020)
		(1002)	(3.93)					
**54**	**Trip-PIM**	334	6.30	-	chloroform	pure	150	[60] (2020)
		(374)	(6.93)					
**55**	**Trip-PBO**	(68)	(8.10)	-	DMAc	pure	-	[65] (2018)
**56**	**Ladder**	(36.5)	(8.50)	45	chloroform	pure	-	[66] (2017)
**Group III**								
**57**	**PAm**	(29.0)	(9.67)	70–80	DMAc	pure	-	[67] (2015)
**58**	**PAm**	(14.2)	(10.2)	70–80	DMAc	pure	-	[68] (2015)
**59**	**PI**	(8.52)	(20.3)	65–75	DMF	pure	-	[69] (2016)

[Note] For the codes, see Chart 4. Data in parentheses are ones before aging.

**Table 5:** Categorization and detailed data for the membrane polymers in Figure 7 (PAs in 2020).

Code	Polymer Type (Subcategory of PA)	*P*_O_2__ (Barrer)	α (=*P*_O_2__/*P*_N_2__)	Thickness (μm)	Casting Solvent	Feed Gas	Ref. (Year)
**1**	**DPA**	12,000	1.13	80–120	toluene	pure	[14] (2008)
		(18,700)	(1.13)				
**2**	**DPA**	10,800	1.24	-	toluene	pure	[15] (2008)
		(14,400)	(1.24)				
**4**	**DPA**	2000	1.84	80	toluene	pure	[17] (2009)
		(4800)	(1.43)				
**5**	**DPA**	1200	1.80	-	toluene	pure	[18] (2006)
		(3500)	(1.80)				
**7**	**DPA**	1000	2.55	30–120	-	mixed	[21] (1993) [14] (2008)
		(1100)	(2.40)				
**8**	**DPA** **(PTMSP)**	150	2.30	-	cyclohexane	pure	[22] (1992)
		(14,800)	(1.29)				
**9**	**DPA**	260	2.70	-	-	pure	[21] (1993)
		(1100)	(2.10)				
**60**	**2DP networked (blend)**	(5320)	(2.45)	30–150	tetrahydrofuran	mixed	[70] (2019)
**61**	**c-c PA (networked)**	(1800)	(2.57)	40–100	chloroform	mixed	[71] (2018)
**62**	**c-c PA (networked)**	(4500)	(2.56)				
**63**	**c-c PA (networked)**	(6950)	(2.44)				
**64**	**DPA**	(3100)	(1.82)	50–80	toluene	pure	[72] (2014)
**65**	**DPA**	(1400)	(2.00)	140–200	toluene	pure	[73] (2017)
**66**	**c-c PA**	(1740)	(2.54)	30–50	chloroform	mixed	[74] (2018)
**67**	**c-c PA**	(1090)	(2.83)				
**68**	**c-c PA**	(405)	(3.00)				
**69**	**c-c PA** **(networked)**	(224)	(4.39)	30–120	tetrahydrofuran	mixed	[75] (2020)
**70**	**2DP (networked)** **(calcd)**	(341)	(4.24)				
**71**	**c-t PA (networked)**	(155)	(4.64)	30–120	toluene	mixed	[76] (2020)
**72**	**c-t PA (networked)**	(141)	(4.92)				
**73**	**c-t PA (hyperbranched)**	(112)	(4.38)	60–120	toluene	mixed	[77] (2017)
**74**	**c-t PA (hyperbranched)**	(106)	(4.46)				
**75**	**c-t PA (hyperbranched)**	(58.7)	(5.00)				
**76**	**c-c PA (multi-stranded)**	(101)	(3.68)	20	toluene	mixed	[78] (2017)
**77**	**c-** **c PA (networked)**	(5.00)	(6.35)	-	-	mixed	[70] (2019)
**78**	**DPA**	(4.00)	(8.90)	-	-	pure	[79] (2015)

[Note] For the codes, see Chart 5. The two polymers (**3** and **6**) are removed from the nine polymers (**1**–**9**) in Table 2, because the aged data for the two polymers were not reported until 2020. Data in parentheses are those before aging.

**Table 6:** Fractional free volume (FFV) and diffusion and dissolution selectivities for selected polymers.

Code	Polymer Type	*P*_O_2__ (Barrer)	FFV	α (*P*_O_2__/*P*_N_2__)	α*_D_* (*D*_O_2__/*D*_N_2__)	α*_S_* (*S*_O_2__/*S*_N_2__)	Ref. (Year)
**8**	**PA** **(PTMSP)**	4860	0.340	1.60	1.47	1.06	[9] (1995)
**11**	**Spiro-PIM** **(PIM-1)**	317		5.00			[25] (2015)
		(2270)	0.26 [30]	(2.80)	2.80	1.00	[24] (2012)
**45**	**Trip-PIM**	3160 (7470)	0.309	4.35	-	-	[44] (2017)
				(3.35)			
**46**	**Trip-PIM**	1170	-	5.40	4.70	1.14	[58] (2015)
		(3290)		(3.60)	(5.00)	(0.71)	
**47**	**Trip-PIM**	1073	-	5.70	5.20	1.10	[59] (2014)
		(2718)		(4.30)	(3.40)	(1.26)	
**50**	**Trip-PIM-PI**	542		6.20	-	-	[61] (2014)
		(627)	-	(5.86)	(5.06)	(1.16)	
**52**	**Trip-PIM**	525	-	5.90	-	-	[44] (2017)
		(368)	-	(6.80)	(6.58)	(1.03)	[63] (2014)
**53**	**Trip-PIM**	500	-	5.21	5.01	1.04	[64] (2020)
		(1002)		(3.93)	(4.24)	(0.95)	
**55**	**Trip-PBO**	(68.0)	-	8.10	6.75	1.18	[65] (2018)
**57**	**PAm**	(29.0)	0.188	9.67	3.88	2.47	[67] (2015)
**58**	**PAm**	(14.2)	0.159	10.2	4.67	2.19	[68] (2015)
**59**	**PI**	(8.52)	0.122	20.3	7.11	2.85	[69] (2016)

[Note] For the codes, see Chart 1 and Chart 4. Data in parentheses are ones before aging.

**Table 7:** Categorization and detailed data for the membrane polymers in Figure 9 (PIs in 2020).

Code	*P*_O_2__ (Barrer)	α (=*P*_O_2__/*P*_N_2__)	Thickness (μm)	Feed Gas	Ref. (Year)
**50**	627	5.86	77	pure	[61] (2014)
**59**	8.52	20.3	65–75	pure	[69] (2016)
**79**	1.67	4.44	200	mixed	[80] (2016)

**Table 8:** Categorization and detailed data for CMSMs in Figure 10 (CMSMs in 2020).

Code	Type of Precursors	Pyrolysis Temperature (°C)	*P*_O_2__ (Barrer)	α (=*P*_O_2__/*P*_N_2__)	Thickness (μm)	Ref. (Year)
**a**	**PI**	550	1530	7.50	50–80	[82] (2010)
**b**	**PI-PIM**	650	1354.5	7.00	45–60	[83] (2019)
			(4.21)	(5.90)		
**c**	**PI-PIM**	600	1071	5.80	80–100	[84] (2013)
**d**	**PI**	500	815	8.20	40–50	[85] (1997)
**e**	**PI**	550	812	7.50	25–60	[86] (2000)
			(69.0)	(4.10)		
**f**	**PI**	500	723.7	24.2	-	[87] (2008)
			(11.6)	(3.90)		
**g**	**PI**	700	707	9.00	25	[88] (2004)
**h**	**Sulfonated phenolic resin**	500	660	12.0	22	[89] (2003)
**i**	**PI**	550	630	10.0	40	[90] (2004)
**j**	**PIM(cross-linked)**	300	582	6.10	50–60	[91] (2011)
**k**	**PI**	700	501	10.0	28	[89] (2004)
**l**	**PI**	650	383	6.70	45–60	[83] (2019)
			(2.46)	(6.50)		
**m**	**PI**	700	256	11.0	28	[89] (2004)
**n**	**PI + ZSM**	650	200	10.3	50	[92] (2008)
**o**	**PI + Ag**	600	170	8.50	20	[93] (2003)
**p**	**PI + MFI zeolite**	600	168	10.0	4.5	[94] (2013)
**q**	**PBI**	580	156	29.1	-	[95] (2014)
			(0.009)	(2.02)		
**r**	**PI**	700	136	15.0	30	[96] (2005)
**s**	**PI**	800	126	8.55	-	[95] (2014)
**t**	**PPO**	290	125(25.0)	10.0(4.50)	25–38	[97] (2007)
**u**	**PI**	800	69	9.02	-	[95] (2014)
**v**	**PI**	800	68	18.5	20–30	[98] (2003)
**w**	**PPO**	650	54.7	11.4	25–38	[97] (2007)
			(7.50)	(5.50)		
**x**	**PI**	800	34.8	11.5	113	[99] (1997)
**y**	**PI**	800	25.0	14.0	35–60	[100] (2003)
**z**	**PI**	800	19.3	32.1	20–30	[98] (2003)
			(0.27)	(4.50)		
**A**	**PI**	900	10.0	21.0	-	[101] (2006)
			(2.00)	(6.62)		
**B**	**PSF**	800	4.50	27.5	-	[102] (2006)
**C**	**PSF**	800	4.43	24.6	50	[103] (2009)
			(0.27)	(3.30)		

[Note] For the codes, see Chart 7.

## Supplementary Materials

The following are available online at https://www.mdpi.com/article/10.3390/polym13173012/s1, S1: Contents of this review.
