# Supplementary material for: Macromolecular Design for Oxygen/Nitrogen Permselective Membranes—Top-Performing Polymers in 2020—
Source: Polymers (Basel). 2021 Sep 6;13(17):3012. doi: 10.3390/polym13173012 (PMC8433776; doi:10.3390/polym13173012)
Supplement: Supplementary file 1 [file polymers-13-03012-s001.zip › polymers-1287250-supplementary.pdf]

Supplementary Materials
for

Macromolecular design for oxygen/nitrogen permselective membranes

– Top-performing polymers in 2020 –

Jianjun Wang, Zhichun Shi, Yu Zang, Hongge Jia, Masahiro Teraguchi, Takashi Kaneko and Toshiki Aoki *

Contents

S1. Contents of this review · S2

Contents

1. Introduction

1.1 Significance of oxygen/nitrogen permselective membranes
1.2 Requirements of polymers as oxygen/nitrogen permselective membranes
1.3 Mechanisms of gas permselective membranes (Figure 1)
1.3.1 Solution-diffusion (SD) mechanism for dense (nonporous) membranes
1.3.2 Molecular-sieving (MS) mechanism for ultramicroporous and microporous membranes
1.3.3 Knudsen (Kd) mechanism for microporous and mesoporous membranes
1.3.4 Transition range between the solution-diffusion (SD) mechanism for dense (nonporous) membranes, the molecular-sieving (MS) mechanism for ultra-microporous membrane, and the Knudsen (Kd) mechanism for microporous and mesoporous membranes
1.4 Robeson's and Pinnau's upper bound lines
1.4.1 Existence of upper bound lines in selectivity-permeability plots
1.4.2 Meaning of the upper bound lines – the slope and intercept
1.4.3 History of the upper bound lines – from 1991 via 2008 to 2015
1.4.4 Aging problems for ultrahigh permeable polymers
1.4.5 Advantage and disadvantages of polymers as materials for oxygen/nitrogen permselective membranes
1.5 Purposes of this review

2 Macromolecular design for oxygen/nitrogen permselective membranes–top-performing polymers

2.1 Top-performing polymers until 2013 as oxygen/nitrogen permselective membranes

2.1.1 Poly(substituted acetylene)s (PAs) (Group I: nos. 1-9 in Figure 3 and Figure 4)

2.1.2 Poly(1-trimethylsilylpropyne) (PTMSP) (an ultrapermeable polymer) (no. 8 in Figure 3 and Figure 4)
2.1.3 Poly(diphenylacetylene)s (PDPAs) (nos.1, 2, 4 - 7, and 9)
2.1.4 Spiro-PIMs (polymers of intrinsic microporosity with spirocarbons) (Group II: nos.10 – 16 in Figure 3 and Chart 1)
(1) Advent of the first PIM (PIM-1) (no.11 in **Figure 3**)
(2) Improved spiro-PIM (nos.10 and 15)
(3) Spiro-PIM with side groups (modified Spiro-PIM) (nos.14 and 16)
(4) Spiro-PIM-PI (nos.12 and 13)

2.1.5 Polyimides (PIs) (Group III: nos.17– 23 in Figure 3 and Figure 5)

2.2 Top-performing polymers in 2020 as oxygen/nitrogen permselective membranes
2.2.1 PDPAs and modified PAs (Group I; nos.1-7 in **Chart 4** and **Figure 6**)
(1) Poly(diphenylacetylene)s (PDPAs)
(2) Modified PAs
2.2.2 Ladder polymers with side aryl groups without spirocarbons and other ladder polymers (Group II; nos.8- 20 in **Chart 4** and **Figure 6**)
(1) Ladder polymers with side aryl groups without spirocarbons (Trip-PIM)
(2) PIs with Trip
(3) Other ladder polymers
(4) Modified Spiro-PIMs
2.2.3 PI or polyamides (Pams) with bulky side groups (Group III, nos.21-23 in **Chart 4** and **Figure 6**)

2.3 Tendency of change in oxygen permeation performance by changing the chemical structures of the polymers

3 Simultaneous improvements of selectivity and permeability of polymer membranes

3.1 Polymer modification by thermal polymer reaction
3.1.1 Thermally rearranged polymers (TR)
3.1.2 Carbon molecular sieve membranes (CMSM)

3.2 Polymer membrane modification by blending with microporous additives – mixed matrix membranes (MMMs).
3.2.1 Required conditions for simultaneous improvements of selectivity and permeability by the MMM method
3.2.2 Types of microporous additives
(1) Zeolites
(2) Metal organic frameworks (MOF)
(3) Microporous organic cages (POC)
3.2.3 Sizes (nanocrystals), shapes (nanosheets), and assembled structures of microporous additives

4 Concluding remarks

4.1. High permeable linear polymers, PTMSP and PIMs

4.2. High permselective inorganic membranes, CMSM and graphene

4.3. Promising membranes from regularly networked organic polymers, 2DP
